# Reconstructed evolutionary history of the yeast septins Cdc11 and Shs1

**DOI:** 10.1093/g3journal/jkaa006

**Published:** 2020-12-07

**Authors:** Julie Takagi, Christina Cho, Angela Duvalyan, Yao Yan, Megan Halloran, Victor Hanson-Smith, Jeremy Thorner, Gregory C Finnigan

**Affiliations:** 1 Department of Molecular and Cell Biology, University of California, Berkeley, Berkeley, CA 94720-3202, USA; 2 Department of Biochemistry and Molecular Biophysics, Kansas State University, Manhattan, KS 66506, USA; 3 Department of Microbiology and Immunology, University of California, San Francisco, CA 94158, USA

**Keywords:** yeast, molecular evolution, septins, cytoskeleton, ancestral gene reconstruction

## Abstract

Septins are GTP-binding proteins conserved across metazoans. They can polymerize into extended filaments and, hence, are considered a component of the cytoskeleton. The number of individual septins varies across the tree of life—yeast (*Saccharomyces cerevisiae*) has seven distinct subunits, a nematode (*Caenorhabditis elegans*) has two, and humans have 13. However, the overall geometric unit (an apolar hetero-octameric protomer and filaments assembled there from) has been conserved. To understand septin evolutionary variation, we focused on a related pair of yeast subunits (Cdc11 and Shs1) that appear to have arisen from gene duplication within the fungal clade. Either Cdc11 or Shs1 occupies the terminal position within a hetero-octamer, yet Cdc11 is essential for septin function and cell viability, whereas Shs1 is not. To discern the molecular basis of this divergence, we utilized ancestral gene reconstruction to predict, synthesize, and experimentally examine the most recent common ancestor (“Anc.11-S”) of Cdc11 and Shs1. Anc.11-S was able to occupy the terminal position within an octamer, just like the modern subunits. Although Anc.11-S supplied many of the known functions of Cdc11, it was unable to replace the distinct function(s) of Shs1. To further evaluate the history of Shs1, additional intermediates along a proposed trajectory from Anc.11-S to yeast Shs1 were generated and tested. We demonstrate that multiple events contributed to the current properties of Shs1: (1) loss of Shs1–Shs1 self-association early after duplication, (2) co-evolution of heterotypic Cdc11–Shs1 interaction between neighboring hetero-octamers, and (3) eventual repurposing and acquisition of novel function(s) for its C-terminal extension domain. Thus, a pair of duplicated proteins, despite constraints imposed by assembly into a highly conserved multi-subunit structure, could evolve new functionality via a complex evolutionary pathway.

## Introduction

Septins comprise a fourth cytoskeletal element, conserved from fungi to metazoans (Pan *et al.* 2007; Nishihama *et al.* 2011; Auxier *et al.* 2019). Each septin contains a GTP-binding fold (G domain) preceded by an N-terminal extension (NTE) of variable length and trailed by a C-terminal extension (CTE) of variable length. A given septin associates with other septins in a defined order into linear hetero-oligomeric complexes, which, in turn, have the capacity to assemble into higher-order structures. Similar to other cytoskeletal components, septin-based structures can adopt unique architectures and geometries *in vivo* and *in vitro*, including linear filaments, arcs, spirals, hourglasses, and rings (Bertin *et al.* 2008, 2012; Garcia *et al.* 2011; Ong *et al.* 2014). Rather than purely contributing to cell shape, septins reportedly have numerous functions in different species, cell types, and subcellular locations. Such functions include: (1) serving as a diffusion barrier tightly associated with the membrane between two distinct cellular compartments (such as in dividing cells, or to separate dendritic spines from the cell body in neurons) (Dobbelaere and Barral 2004; Caudron and Barral 2009), (2) sensing membrane curvature (Bridges *et al.* 2016; Cannon *et al.* 2019), and (3) acting as a platform for recruitment of septin-associated proteins for information exchange via signaling pathways (Neubauer and Zieger 2017; Perez *et al.* 2016). Many of these functions have been conserved across eukaryotes; importantly, septin dysfunction in humans has been linked to a number of diseases, including male infertility, cancer, and neurodegenerative diseases (Shen *et al.* 2017; Wang *et al.* 2018; Xu *et al.* 2018; Marcus *et al.* 2019).

Early studies on septins focused on the unicellular eukaryote *Saccharomyces cerevisiae*. In this yeast, seven genes encoding distinct septins were identified—*CDC3, CDC10, CDC11, CDC12, SHS1, SPR3*, and *SPR28—*the latter two are only expressed and functional during sporulation (Kaback and Feldberg 1985; Ozsarac *et al.* 1995; De Virgilio *et al.* 1996; Garcia *et al.* 2016; Heasley and McMurray 2016). Disruption of *CDC3, CDC10, CDC11*, or *CDC12* prevented completion of cytokinesis and resulted in cell death (Hartwell 1978); labeling experiments later determined that the cognate proteins localize to the division site (bud neck) between a mother and daughter cell undergoing mitosis and form a complex 3D super-structure there (Byers and Goetsch 1976; Haarer and Pringle 1987; Cid *et al.* 1998; Bertin *et al.* 2012). Extensive genetic and biochemical approaches determined that two copies of each of the four essential septins form a linear apolar hetero-octamer with a twofold axis of symmetry (Cdc11–Cdc12–Cdc3–Cdc10–Cdc10–Cdc3–Cdc12–Cdc11), that hetero-octamers polymerize end-to-end via Cdc11–Cdc11 interaction to form long, laterally paired filaments, and that formation of filaments is essential for septin function *in vivo* (Bertin *et al.* 2008; McMurray *et al.* 2011). Subsequent work showed that the fifth mitotically expressed septin, Shs1, could also occupy the terminal position, thus forming Shs1–Cdc12–Cdc3–Cdc10–Cdc10–Cdc3–Cdc12–Shs1 hetero-octamers (Garcia *et al.* 2011; McMurray *et al.* 2011; Bertin *et al.* 2012; Booth *et al.* 2015; Finnigan, Takagi, *et al.* 2015). However, it is clear that there are significant functional differences between Cdc11 and Shs1; the former subunit is essential for filament formation and viability *in vivo*, whereas Shs1 is non-essential under many standard growth conditions (Iwase *et al.* 2007; Garcia *et al.* 2011). Use of sensitized genetic backgrounds, structural data, and biochemical assays revealed certain unique roles for Shs1 within *S. cerevisiae* and related fungal species that influence filament curvature and/or assembly state, association with the plasma membrane, and coordinated recruitment of non-septin binding partners, such as the myosin-binding protein Bni5 (Egelhofer *et al.* 2008; Buttery *et al.* 2012; Meseroll *et al.* 2012, 2013; Booth *et al.* 2015; Finnigan, Booth, *et al.* 2015; Finnigan, Takagi, *et al.* 2015).

The CTEs of both Cdc3 and Cdc12 were found to participate in coiled coil (CC) interactions that serve as cross-bracing within each hetero-octamer and that also provide contacts responsible for the lateral pairing of septin filaments (Versele *et al.* 2004). As the cell cycle proceeds, the hourglass-shaped septin-based collar-like structure at the bud neck undergoes a transition to a split (double ring) structure concomitant with the onset of cytokinesis (Bertin *et al.* 2008, 2012; Garcia *et al.* 2011; McMurray *et al.* 2011).

Within each hetero-octamer, there are alternating interfaces between neighboring subunits deduced from crystallized septin complexes: the G interface, in which the GTP/GDP-binding pockets in each subunit face each other; and the NC interface, wherein helical elements within the N- and C-terminal sequences that are proximal and distal, respectively, to the G domain face each other (Sirajuddin *et al*. 2007, 2009; Ong *et al.* 2014; Brausemann *et al.* 2016). In a hetero-octamer, the central Cdc10–Cdc10 pair associates via an NC interaction, whereas each Cdc10 associates with its flanking Cdc3 via a G interface, and so forth.

Across eukaryotes (with the exception of higher plants, which lack septins), the number of septin subunits varies—for example, one in the green alga *Chlamydomonas reinhardtii*, two in the nematode *Caenorhabditis elegans*, five in the fruit fly *Drosophila. melanogaster*, and 13 in *Homo sapiens*, which are differentially expressed in specific cell types and tissues (Field *et al.* 1996; Adam *et al.* 2000; Nguyen *et al.* 2000; Kinoshita 2003; John *et al.* 2007; Cao *et al.* 2009; Nishihama *et al.* 2011; Pinto *et al.* 2017). However, the hetero-octameric complex with distinct subunits occupying specific positions within the structure has been conserved from yeast to humans (Bertin *et al.* 2008; McMurray and Thorner 2019; Mendonca *et al.* 2019; Soroor *et al.* 2020). Phylogenetic analyses indicate that during fungal and metazoan evolution gene duplications gave rise to the current repertoire of septin subunits (Pan *et al.* 2007; Nishihama *et al.* 2011; Auxier *et al.* 2019). Such increases in biological complexity across deep evolutionary time wherein a multi-subunit complex acquires additional functional components through gene duplication and divergence have clearly occurred in other instances, including the V-type ATPase (Finnigan *et al.* 2011, 2012), the proteasome (Wollenberg and Swaffield 2001), the TRiC/CCT chaperonin (Gestaut *et al.* 2019), and the NADH:ubiquinone oxidoreductase (Gabaldon *et al.* 2005). However, how inclusion of a newly duplicated protein within an existing multi-protein ensemble occurs is more challenging to explain for a non-essential subunit (such as Shs1 in the yeast septin hetero-octamer) that has been maintained rather than pseudogenized and lost.

In addition, the molecular evolution of two subunits, both occupying the same position within a complex structure, presents a number of biochemical constraints. In the case of the terminal septin subunits, both Cdc11 and Shs1 must retain the ability to bind guanine nucleotide as well as the capacity to associate with the penultimate subunit Cdc12 via a G interface. On the other hand, whether to preserve the capacity for homotypic NC interface interaction, which supports formation of paired linear filaments (as exhibited by Cdc11), or to evolve the capacity for heterotypic interaction (such as exhibited by Shs1- and Cdc11-capped hetero-octamers) and thereby acquire the capacity to form more complex geometric arrangements in higher order structures leaves room for why the advent of Shs1 may have provided some selective advantage.

Viewed in this light, Cdc11 and Shs1 provide a unique opportunity to conduct an analysis grounded in evolutionary principles to address questions relating to how a new subunit arising from the duplication of a pre-existing one is first tolerated, retains the capacity for integration into a complex structure, and diverges to confer new properties without disrupting essential functions. Understanding how protein complexes have increased in complexity through evolutionary time remains a critical task for multiple fields of study. A detailed mechanistic history of how protein complexes, protein–protein interfaces, and specific protein domains evolve can provide not only a proper, “vertical” historical context for current day experimental comparisons of existing proteins (Merkl and Sterner 2016), but may someday have predictive power for understanding protein evolution within rapidly evolving species such as micro-organisms.

Toward these ends, in this study, we utilized ancestral gene reconstruction (Thornton 2004) (AGR) to predict, generate, and test in modern *S. cerevisiae* cells the assembly, localization, and function(s) of the pre-duplicated ancestral subunit (termed “Anc.11-S”) of Cdc11 and Shs1, as well as four additional ancestors and three modern fungal septins. Our study determined that Anc.11-S can partially replace modern Cdc11 in yeast yet was unable to form productive heterotypic interfaces with Shs1. Furthermore, all tested ancestral and fungal septins seemed to be able to associate with Cdc12 through the G interface, albeit with very different apparent affinities. Evolution of the Shs1 subunit involves multiple distinct changes including early loss of homotypic Shs1–Shs1 interactions, development of a distinct G domain, development of an optimized Cdc11–Shs1 heterotypic interaction, and very recent evolution of a modern function of its CTE. These findings are the first to highlight the complex evolution of a unique pair of essential/non-essential components in a highly conserved multi-protein complex.

## Materials and methods

### *In silico* reconstruction of ancestral protein sequences

Putative septin orthologs of budding yeast Cdc11 or Shs1 within the fungal kingdom were identified using BLAST (NCBI); these are listed in Supplementary Table S1. Sequences were aligned using three separate methods: MUSCLE (Edgar 2004), MSAprobs (Liu *et al.* 2010), and PRANK (Loytynoja and Goldman 2005, 2008). For each alignment, ancestral protein sequences for all shared ancestors were inferred with maximum-likelihood phylogenetics, using PAML (Yang 2007) and PhyloBot (Hanson-Smith and Johnson 2016). All three approaches (Supplementary Figure S1) yielded a consensus sequence for Anc.11-S, with differences concentrated within the CTE domain. We chose to experimentally assay the ancestral sequences from the MUSCLE approach, as the total length of the protein was the longest of the three (418 residues) indicating that MUSCLE yielded the most conservative alignment of the three approaches. The posterior probabilities (PPs) from reconstructed sequences are summarized in Supplementary Table S2. For each ancestral gene, a set of residues with PP scores below a determined threshold were randomly sampled and individually tested *in vivo* compared to the original reconstruction; these findings will be presented in a separate manuscript.

### Yeast strains and plasmids

*Saccharomyces cerevisiae* strains used in this study can be found in Table 1 and Supplementary Table S3 and plasmids used in this study can be found in Table 2. Reconstructed ancestral genes were generated by custom gene synthesis (Genscript) using a yeast codon bias and carried in plasmid pUC57. For all constructs, *in vivo* plasmid assembly (Finnigan and Thorner 2015) was used to link together the necessary DNA components (promoter, coding regions, tags, terminators, and selection cassettes). A modified polymerase chain reaction (PCR)-based mutagenesis protocol (Zheng *et al.* 2004) was used to introduce substitutions prior to assembly. Briefly, a *CEN*-based plasmid was digested with a unique restriction site downstream of a cloned promoter sequence and co-transformed into yeast (standard lithium acetate-based protocol) (Gietz and Schiestl 2007) with the necessary amplified PCR fragments containing homology to adjacent sequences. Typically, a downstream drug-resistance cassette (Goldstein and McCusker 1999) was also included for additional selection purposes and for use in one-step chromosomal integration strategies. Placement of DNA constructs at the required genomic loci utilized upstream promoter sequence as well as the common MX-based terminator sequence present on selection cassettes. This “marker swapping” technique allowed for the integration of the entire gene fusion. Given that there is still the possibility for the marker cassette to swap without integration of the upstream sequence (using the identical MX promoter sequences), all integrations were confirmed using diagnostic PCRs to confirm the presence of the desired integrated DNA construct in addition to the switch in selection marker. Following *in vivo* plasmid assembly, constructs were confirmed further using either in-house (UC Berkeley DNA Sequencing Facility) or commercial (Genscript) Sanger DNA sequencing. Following chromosomal integration, modified loci were amplified by PCR, purified, and sequenced (Genscript). Sequences of all the DNAs used in this study can be found in Supplementary Figure S2.

**Table 1:** Yeast strains used in this study

Strain	Genotype	Reference
BY4741	*MAT* **a** *his3Δ1 leu2Δ0 met15Δ0 ura3Δ0*	Brachmann *et al.* (1998)
GFY-6^a^^,^^*b*^	BY4741; *cdc10Δ::S.c.CDC10::mCherry::ADH1(t)::S.p.HIS5 shs1ΔKan^R^* + pJT2022	Finnigan, Takagi, *et al.* (2015)
GFY-38	BY4741; *shs1Δ::Kan^R^*	Finnigan, Takagi, *et al.* (2015)
GFY-58^c^	BY4741; *cdc11Δ::S.c.CDC11::mCherry::ADH1(t)::S.p.HIS5*	Finnigan, Takagi, *et al.* (2015)
GFY-87^d^	BY4741; *cdc10Δ::Kan^R^ shs1Δ::S.c.SHS1::GFP::ADH1(t)::Nat^R^+* pJT2022	Finnigan, Takagi, *et al.* (2015)
GFY-137	BY4741; *cdc10Δ::Kan^R^ shs1Δ::Hyg^R^+* pJT2022	Finnigan, Takagi, *et al.* (2015)
GFY-93	BY4741; *cdc10Δ::Kan^R^ shs1Δ::S.c.shs1(Δ2-18)::GFP::Nat^R^* + pJT2022	Finnigan, Takagi, *et al.* (2015)
GFY-139	BY4741; *cdc12Δ::S.c.cdc12(K391N Δ392-407)::ADH1(t)::Hyg^R^ shs1Δ::Kan^R^+* pJT1622	Finnigan, Takagi, *et al.* (2015)
GFY-147	BY4741; *cdc11Δ::Kan^R^ shs1Δ::S.c.SHS1::GFP::ADH1(t)::Nat^R^+* pJT1520	Finnigan, Takagi, *et al.* (2015)
GFY-153	BY4741; *cdc11Δ::Kan^R^*+ pJT1520	This study
GFY-123	BY4741; *cdc11Δ::S.c.cdc11(Δ2-18)::mCherry::S.p.HIS5*+ pJT1520	Finnigan, Takagi, *et al.* (2015)
GFY-165	BY4741; *cdc11Δ::S.c.cdc11(Δ2-18Δ)::mCherry::S.p.HIS5 shs1Δ::Hyg^R^*+ pJT1520	Finnigan, Takagi, *et al.* (2015)
GFY-161	BY4741; *cdc11Δ::S.c.cdc11(Δ2-18)::mCherry::S.p.HIS5 shs1Δ::S.c.shs1(Δ2-18)::GFP::Nat^R^*+ pJT1520	Finnigan, Takagi, *et al.* (2015)
GFY-160	BY4741; *cdc11Δ::S.c.CDC11::mCherry::ADH1(t)::S.p.HIS5 shs1Δ::S.c.SHS1::GFP::ADH1(t)::Nat^R^*+ pJT1520	Finnigan, Takagi, *et al.* (2015)
GFY-163	BY4741; *cdc11Δ::Kan^R^ shs1Δ::Hyg^R^*+ pJT1520	Finnigan, Takagi, *et al.* (2015)
GFY-164	BY4741; *cdc11Δ::S.c.CDC11::mCherry::ADH1(t)::S.p.HIS5 shs1Δ::Hyg^R^*+ pJT1520	Finnigan, Takagi, *et al.* (2015)
GFY-293	BY4741; *cdc11Δ::S.c.cdc11(Δ357-415)::mCherry::ADH1(t)::S.p.HIS5 shs1Δ::S.c.SHS1::GFP::ADH1(t)::Nat^R^* + pJT1520	Finnigan, Takagi, *et al.* (2015)
GFY-302	BY4741; *cdc12Δ::S.c.cdc12(K391N Δ392-407)::ADH1(t)::Hyg^R^ shs1Δ::S.c.SHS1::GFP::ADH1(t)::Nat^R^*+ pJT1622	Finnigan, Takagi, *et al.* (2015)
GFY-437	BY4741; *cdc11Δ::Nat^R^ shs1Δ::Hyg^R^ cdc12Δ::S.c.cdc12(W267A)::ADH1(t)::Kan^R^*+ pJT1520/pJT1622	Finnigan, Takagi, *et al.* (2015)
GFY-476	BY4741; *cdc11Δ::Kan^R^ shs1Δ::Anc.S::GFP::ADH1(t)::Nat^R^* + pJT1520	This study
GFY-477	BY4741; *cdc12Δ::S.c.cdc12(K391N Δ392-407)::ADH1(t)::Hyg^R^ shs1Δ::Anc.S::GFP::ADH1(t)::Nat^R^* + pJT1622	This study
GFY-478	BY4741; *cdc12Δ::S.c.cdc12(K391N Δ392-407)::ADH1(t)::Hyg^R^ shs1Δ::Anc.11-S::GFP::ADH1(t)::Nat^R^* + pJT1622	This study
GFY-479	BY4741; *cdc11Δ::Anc.11-S::GFP::ADH1(t)::Nat^R^* + pJT1520	This study
GFY-480	BY4741; *cdc11Δ::Anc.11-S::GFP::ADH1(t)::Nat^R^ shs1Δ::Hyg^R^* + pJT1520	This study
GFY-481	BY4741; *cdc11Δ::Anc.11::mCherry::ADH1(t)::S.p.HIS5 *+* *pJT1520	This study
GFY-482	BY4741; *cdc11Δ::Anc.11::mCherry::ADH1(t)::S.p.HIS5 shs1Δ::Hyg^R^* + pJT1520	This study
GFY-483	BY4741; *cdc11Δ::Kan^R^ shs1Δ::Anc.11-S::GFP::ADH1(t)::Nat^R^* + pJT1520	This study
GFY-485	BY4741; *cdc11Δ::S.c.cdc11(Δ357-415)::mCherry::ADH1(t)::S.p.HIS5 shs1Δ::Anc.11-S::GFP::ADH1(t)::Nat^R^* + pJT1520	This study
GFY-486	BY4741; *cdc11Δ::S.c.cdc11(Δ357-415)::mCherry::ADH1(t)::S.p.HIS5 shs1Δ::Anc.S::GFP::ADH1(t)::Nat^R^* + pJT1520	This study
GFY-564	BY4741; *cdc11Δ::S.c.cdc11(Δ2-18)::mCherry::ADH1(t)::S.p.HIS5 shs1Δ::Anc.11-S(Δ2-18)::GFP::ADH1(t)::Nat^R^* + pJT1520	This study
GFY-566	BY4741; *cdc11Δ::Anc.11-S(Δ2-18)::GFP::ADH1(t)::Nat^R^* + pJT1520	This study
GFY-582	BY4741; *cdc11Δ::Anc.11(Δ2-17)::mCherry::ADH1(t)::S.p.HIS5 shs1Δ::Hyg^R^* + pJT1520	This study
GFY-583	BY4741; *cdc11Δ::Kan^R^ shs1Δ::Anc.S(1-344)::S.c.SHS1(349-551)::GFP::ADH1(t)::Nat^R^* + pJT1520	This study
GFY-584	BY4741; *cdc11Δ::Kan^R^ shs1Δ::Anc.11-S(1-307)::S.c.SHS1(349-551)::GFP::ADH1(t)::Nat^R^* + pJT1520	This study
GFY-586	BY4741; *cdc11Δ::Anc.11(Δ2-17)::mCherry::ADH1(t)::Sp-HIS5 *+* *pJT1520	This study
GFY-637	BY4741; *cdc11Δ::Kan^R^ shs1Δ::A.g.SHS1(1-335)::S.c.SHS1(340-551)::eGFP::ADH1(t)::Nat^R^* + pJT2022	Finnigan, Takagi, *et al.* (2015)
GFY-639	BY4741; *cdc11Δ::Kan^R^ shs1Δ::S.c.SHS1(1-339)::A.g.SHS1(336-580)::GFP::ADH1(t)::Nat^R^* + pJT1520	Finnigan, Takagi, *et al.* (2015)
GFY-643	BY4741; *cdc10Δ::Kan^R^ shs1Δ::A.g.SHS1(1-580)::GFP::ADH1(t)::Nat^R^* + pJT2022	Finnigan, Takagi, *et al.* (2015)
GFY-644	BY4741; *cdc10Δ::Kan^R^ shs1Δ::S.c.SHS1(1-339)::A.g.SHS1(336-580)::eGFP::ADH1(t)::Nat^R^* + pJT2022	Finnigan, Takagi, *et al.* (2015)
GFY-650	BY4741; *cdc10Δ::Kan^R^ shs1Δ::Anc.11-S(1-307)::S.c.SHS1(349-551)::GFP::ADH1(t)::Nat^R^ +* pJT2022	This study
GFY-653	BY4741; *cdc10Δ::Kan^R^ shs1Δ::Anc.S(1-344)::S.c.SHS1(349-551)::GFP::ADH1(t)::Nat^R^* + pJT1520	This study
GFY-655	BY4741; *cdc10Δ::Kan^R^ shs1Δ::A.g.SHS1(1-335)::S.c.SHS1(340-551)::eGFP::ADH1(t)::Nat^R^* + pJT2022	Finnigan, Takagi, *et al.* (2015)
GFY-660	BY4741; *cdc11Δ::Anc.11-S(Δ2-18)::GFP::ADH1(t)::Nat^R^ shs1Δ::Hyg^R^* + pJT1520	This study
GFY-683	BY4741; *cdc11Δ::Kan^R^ shs1Δ::A.g.SHS1(1-580)::GFP::ADH1(t)::Nat^R^* + pJT1520	Finnigan, Takagi, *et al.* (2015)
GFY-695	BY4741; *cdc11Δ::Anc.11-S(G30D)::GFP::ADH1(t)::Nat^R^ shs1Δ::Hyg^R^* + pJT1520	This study
GFY-718	BY4741; *cdc11Δ::S.c.cdc11(G29D)::mCherry::ADH1(t)::Kan^R^ shs1Δ::Anc.11-S(G30D)::GFP::ADH1(t)::Nat^R^* + pJT1520	This study
GFY-760	BY4741; *cdc10Δ::Kan^R^ shs1Δ::Anc.S::GFP::ADH1(t)::Nat^R^* + pJT2022	This study
GFY-763	BY4741; *cdc10Δ::Kan^R^ shs1Δ::Anc.S1::GFP::ADH1(t)::Nat^R^* + pJT2022	This study
GFY-765	BY4741; *cdc10Δ::Kan^R^ shs1Δ::Anc.S2::GFP::ADH1(t)::Nat^R^* + pJT2022	This study
GFY-815	BY4741; *cdc10Δ::Kan^R^ shs1Δ::Anc.11-S::GFP::ADH1(t)::Nat^R^ +* pJT2022	This study
GFY-860	BY4741; *cdc11Δ::Kan^R^ shs1Δ::S.c.SHS1(1-348)::Anc.11-S(308-418)::GFP::ADH1(t)::Nat^R^* + pJT1520	This study
GFY-862	BY4741; *cdc10Δ::Kan^R^ shs1Δ::Anc.S1(1-353)::S.c.SHS1(349-551)::GFP::ADH1(t)::Nat^R^* + pJT2022	This study
GFY-864	BY4741; *cdc11Δ::Kan^R^ shs1Δ::S.c.SHS1(1-348)::Anc.S(345-534)::GFP::ADH1(t)::Nat^R^* + pJT1520	This study
GFY-867	BY4741; *cdc10Δ::Kan^R^ shs1Δ::Anc.S2(1-347)::S.c.SHS1(349-551)::GFP::ADH1(t)::Nat^R^* + pJT2022	This study
GFY-868	BY4741; *cdc10Δ::Kan^R^ shs1Δ::S.c.SHS1(1-348)::Anc.S2(348-542)::GFP::ADH1(t)::Nat^R^* + pJT2022	This study
GFY-869	BY4741; *cdc10Δ::Kan^R^ shs1Δ::S.c.SHS1(1-348)::Anc.S(345-534)::GFP::ADH1(t)::Nat^R^* + pJT2022	This study
GFY-874	BY4741; *cdc11Δ::Kan^R^ shs1Δ::S.c.SHS1(1-348)::Anc.S2(348-542)::GFP::ADH1(t)::Nat^R^* + pJT1520	This study
GFY-876	BY4741; *cdc11Δ::Kan^R^ shs1Δ::Anc.S2(1-347)::S.c.SHS1(349-551)::GFP::ADH1(t)::Nat^R^* + pJT2022	This study
GFY-878	BY4741; *cdc11Δ::Kan^R^ shs1Δ::Anc.S1(1-353)::S.c.SHS1(349-551)::GFP::ADH1(t)::Nat^R^* + pJT1520	This study
GFY-879	BY4741; *cdc10Δ::Kan^R^ shs1Δ::S.c.SHS1(1-348)::Anc.S1(354-558)::GFP::ADH1(t)::Nat^R^* + pJT2022	This study
GFY-881	BY4741; *cdc10Δ::Kan^R^ shs1Δ::S.c.SHS1(1-348)::Anc.11-S(308-418)::GFP::ADH1(t)::Nat^R^* + pJT2022	This study
GFY-893	BY4741; *cdc11Δ::Kan^R^ shs1Δ::S.c.SHS1(1-348)::Anc.S1(354-558)::GFP::ADH1(t)::Nat^R^* + pJT1520	This study
GFY-922	BY4741; *cdc11Δ::Kan^R^ shs1Δ::C.a.SHS1(1-366)::S.c.SHS1(349-551)::GFP::ADH1(t)::Nat^R^* + pJT1520	This study
GFY-925	BY4741; *cdc11Δ::Kan^R^ shs1Δ::C.g.SHS1(1-367)::S.c.SHS1(349-551)::GFP::ADH1(t)::Nat^R^* + pJT1520	This study
GFY-926	BY4741; *cdc11Δ::Kan^R^ shs1Δ::C.a.SHS1::GFP::ADH1(t)::Nat^R^* + pJT1520	This study
GFY-929	BY4741; *cdc11Δ::Kan^R^ shs1Δ::S.c.SHS1(1-348)::C.g.SHS1(368-533)::GFP::ADH1(t)::Nat^R^* + pJT1520	This study
GFY-931	BY4741; *cdc10Δ::Kan^R^ shs1Δ::C.g.SHS1::GFP::ADH1(t)::Nat^R^* + pJT2022	This study
GFY-935	BY4741; *cdc10Δ::Kan^R^ shs1Δ::S.c.SHS1(1-348)::C.g.SHS1(368-533)::GFP::ADH1(t)::Nat^R^* + pJT2022	This study
GFY-936	BY4741; *cdc10Δ::Kan^R^ shs1Δ::S.c.SHS1(1-348)::C.a.SHS1(367-666)::GFP::ADH1(t)::Nat^R^* + pJT2022	This study
GFY-938	BY4741; *cdc11Δ::Kan^R^ shs1Δ::S.c.SHS1(1-348)::C.a.SHS1(367-666)::GFP::ADH1(t)::Nat^R^* + pJT1520	This study
GFY-939	BY4741; *cdc11Δ::Kan^R^ shs1Δ::Anc.S1::GFP::ADH1(t)::Nat^R^* + pJT1520	This study
GFY-940^e^	BY4741; *cdc10Δ::Kan^R^ shs1Δ::C.a.SHS1::GFP::ADH1(t)::Nat^R^* + pJT2022	This study
GFY-943	BY4741; *cdc11Δ::Kan^R^ shs1Δ::C.g.SHS1::GFP::ADH1(t)::Nat^R^* + pJT1520	This study
GFY-944	BY4741; *cdc11Δ::Kan^R^ shs1Δ::Anc.S2::GFP::ADH1(t)::Nat^R^* + pJT1520	This study
GFY-948	BY4741; *cdc10Δ::Kan^R^ shs1Δ::C.g.SHS1(1-367)::S.c.SHS1(349-551)::GFP::ADH1(t)::Nat^R^* + pJT2022	This study
GFY-949	BY4741; *cdc10Δ::Kan^R^ shs1Δ::C.a.SHS1(1-366)::S.c.SHS1(349-551)::GFP::ADH1(t)::Nat^R^* + pJT1520	This study
GFY-974	BY4741; *cdc11Δ::Anc.S::mCherry::ADH1(t)::S.p.HIS5 shs1Δ::Hyg^R^* + pJT1520	This study
GFY-975	BY4741; *cdc11Δ::Anc.S::mCherry::ADH1(t)::S.p.HIS5 *+* *pJT1520	This study
GFY-1023	BY4741; *cdc11Δ::Anc.11(Δ2-17)::mCherry::ADH1(t)::S.p.HIS5 shs1Δ::S.c.shs1(Δ2-18)::GFP::ADH1(t)::Nat^R^* + pJT1520	This study
GFY-1025	BY4741; *cdc11Δ::Anc.11-S(Δ2-18)::mCherry::ADH1(t)::S.p.HIS5 shs1Δ::S.c.shs1(Δ2-18)::GFP::ADH1(t)::Nat^R^* + pJT1520	This study
GFY-1026	BY4741; *cdc11Δ::Anc.11-S(G30D)::mCherry::ADH1(t)::S.p.HIS5 shs1Δ::S.c.shs1(G30D)::GFP::ADH1(t)::Nat^R^* + pJT1520	This study

a For clarity, selection cassettes (*e.g.* Nat^R^) only indicate the gene of interest; all are flanked by the commonly used *prMX* and *MX(t)* sequences.

b Abbreviations of the fungal genus and species are included for all modified genes: *S.c.*, *Saccharomyces cerevisiae*, *C.g.*, *Candida glabrata*, *C.a.*, *Candida albicans*, *A.g.*, *Ashbya gossypii*, and *S.p.*, *Schizosaccharomyces pombe.*

c The *URA3*-based covering vector (expressing WT *CDC11*) was removed by multiple rounds of selection on medium containing 5-FOA.

d The GFP sequence used in these fusions includes an N-terminal linker sequence of GRRIPGLIN as well as F64L and S65T substitutions.

e The *C. albicans* protein is missing the residue N613 (which exists within a stretch of consecutive Asn residues) within this strain. The total septin protein size is therefore expected to be 665 amino acids, not 666.

**Table 2:** Plasmids used in this study

Plasmid	Description	Reference
pRS315	*CEN*, *LEU2*	Sikorski and Hieter (1989)
pJT2022	YCplac33 *URA3 CDC10*	McMurray *et al.* (2011)
pJT1622	YCplac33 *URA3 CDC12*	Versele *et al.* (2004)
pJT1520	pRS316 *URA3 CDC11*	Versele *et al.* (2004)
pGF-IVL-159^a^	pRS315; *prCDC11::Anc.11-S::GFP::ADH1(t)::Nat^R^*	This study
pGF-IVL-168	pRS315; *prCDC11::Anc.S::GFP::ADH1(t)::Nat^R^*	This study
pGF-preIVL-59^b^	pRS315; *prSHS1::S.c.SHS1::GFP::ADH1(t)::Nat^R^*	Finnigan, Takagi, *et al.* (2015)
pGF-IVL-286^c^	pRS315; *prGAL1/10::S.c.SHS1::GFP::ADH1(t)::Kan^R^*	This study
pGF-IVL-287^d^	pRS315; *prGAL1/10::S.c.CDC11::mCherry::ADH1(t)::Kan^R^*	This study
pGF-IVL-1278	pRS315; *prGAL1/10::Anc.11-S::GFP::ADH1(t)::Kan^R^*	This study
pGF-IVL-1279	pRS315; *prGAL1/10::Anc.11::mCherry::ADH1(t)::Kan^R^*	This study
pGF-IVL-1280	pRS315; *prGAL1/10::Anc.S::GFP::ADH1(t)::Kan^R^*	This study
pGF-IVL-1281	pRS315; *prGAL1/10::Anc.S1::GFP::ADH1(t)::Kan^R^*	This study
pGF-IVL-1282	pRS315; *prGAL1/10::Anc.S2::GFP::ADH1(t)::Kan^R^*	This study
pGF-IVL-1283	pRS315; *prGAL1/10::C.g.SHS1::GFP::ADH1(t)::Kan^R^*	This study
pGF-IVL-1284	pRS315; *prGAL1/10::C.a.SHS1::GFP::ADH1(t)::Kan^R^*	This study
pGF-IVL-1285	pRS315; *prGAL1/10::A.g.SHS1::GFP::ADH1(t)::Kan^R^*	This study
pGF-IVL-1286^e^	pRS315; *prGAL1/10::Anc.11-S(G30D)::GFP::ADH1(t)::Kan^R^*	This study
pGF-IVL-1287	pRS315; *prGAL1/10::Anc.11(G29D)::mCherry::ADH1(t)::Kan^R^*	This study
pGF-IVL-1288	pRS315; *prGAL1/10::Anc.S (G37D)::GFP::ADH1(t)::Kan^R^*	This study
pGF-IVL-1289	pRS315; *prGAL1/10::Anc.S1 (G37D)::GFP::ADH1(t)::Kan^R^*	This study
pGF-IVL-1290	pRS315; *prGAL1/10::Anc.S2(G30D)::GFP::ADH1(t)::Kan^R^*	This study
pGF-IVL-1291	pRS315; *prGAL1/10::C.g.shs1(G29D)::GFP::ADH1(t)::Kan^R^*	This study
pGF-IVL-1292	pRS315; *prGAL1/10::C.a.shs1(G43D)::GFP::ADH1(t)::Kan^R^*	This study
pGF-IVL-1293	pRS315; *prGAL1/10::A.g.shs1(G33D)::GFP::ADH1(t)::Kan^R^*	This study
pGF-IVL-1343	pRS315; *prGAL1/10::S.c.shs1(G30D)::GFP::ADH1(t)::Kan^R^*	This study
pGF-IVL-1344	pRS315; *prGAL1/10::S.c.cdc11(G29D)::mCherry::ADH1(t)::Kan^R^*	This study

a The GFP sequence used in these fusions includes an N-terminal linker sequence of GRRIPGLIN as well as F64L and S65T substitutions. Ancestral (abbreviated “Anc”) genes were synthesized *de novo* with a yeast codon bias. The commonly used *prMX* and *MX(t)* sequences were included flanking all drug resistance cassettes (*e.g.* Nat^R^).

b Abbreviations of the fungal genus and species are included for *SHS1* and *CDC11* genes: *S.c.*, *Saccharomyces cerevisiae*, *C.g.*, *Candida glabrata*, *C.a.*, *Candida albicans*, and *A.g.*, *Ashbya gossypii*. The *S.c.SHS1* gene has a silent substitution within codon 314 (Glycine).

c The *GAL1/10* promoter included 814 base pairs of 5′ UTR.

d Translational fusions to mCherry (235 amino acids) did not include any linker sequence.

e Gly-to-Asp substitutions within the (putative) P-loop were generated based on alignments to *Sc-SHS1* for fungal orthologs and ancestral genes.

### Culture conditions

Budding yeast strains were grown on 2% agar plates or in liquid culture (in a temperature-controlled floor shaker). Rich media (YPD) consisted of 2% peptone, 1% yeast extract, and 2% dextrose. Synthetic media included yeast trace nutrients, amino acids, and ammonium sulfate. All sugar solutions (final concentrations included 2% dextrose, 2% galactose, 2% raffinose with 0.2% sucrose) were filter sterilized (not autoclaved). Plates contained 0.5 g/l of 5-fluoro-orotic acid (5-FOA) (to eliminate potential contaminants, the 5-FOA solution was heated to 70°C for 30 min before being cooled and filtered).

### Fluorescence microscopy

All plasmid-carrying strains were selected by streaking for single colonies at least twice on agar plates. Cultures were grown overnight at 30°C, back diluted into rich medium for 4.0 or 4.5 h at 30°C, harvested, and examined within 30 min at room temperature under a fluorescence microscope (Leica, model DMI6000; Leica Microsystems, Buffalo Grove, IL, USA), equipped with a 100× lens and appropriate cutoff filters for visualization of GFP and mCherry (monomeric red fluorescent protein derivative) fluorescence (Semrock), and images were acquired using a Leica DFC340 FX camera. Image capture and analysis was performed using software from the Leica Microsystems Application Suite and ImageJ (Schindelin *et al.* 2015). Images were captured using identical exposure times and evaluated in a single-blind manner. Representative images for each strain are shown and rescaled in the same way; adjustment of contrast was done per individual image.

### Data availability

The authors will make available the reagents (DNA plasmids or yeast strains) and/or datasets used to confirm the conclusions of this manuscript upon reasonable request. A Supplementary file S1 is available at FigShare and contains DNA sequences used, additional tables, and additional figures. 

Supplementary material is available at figshare DOI: https://doi.org/10.25387/g3.13205906

## Results

### Evolution of the terminal septins Cdc11 and Shs1 within fungi

**Figure 1:**
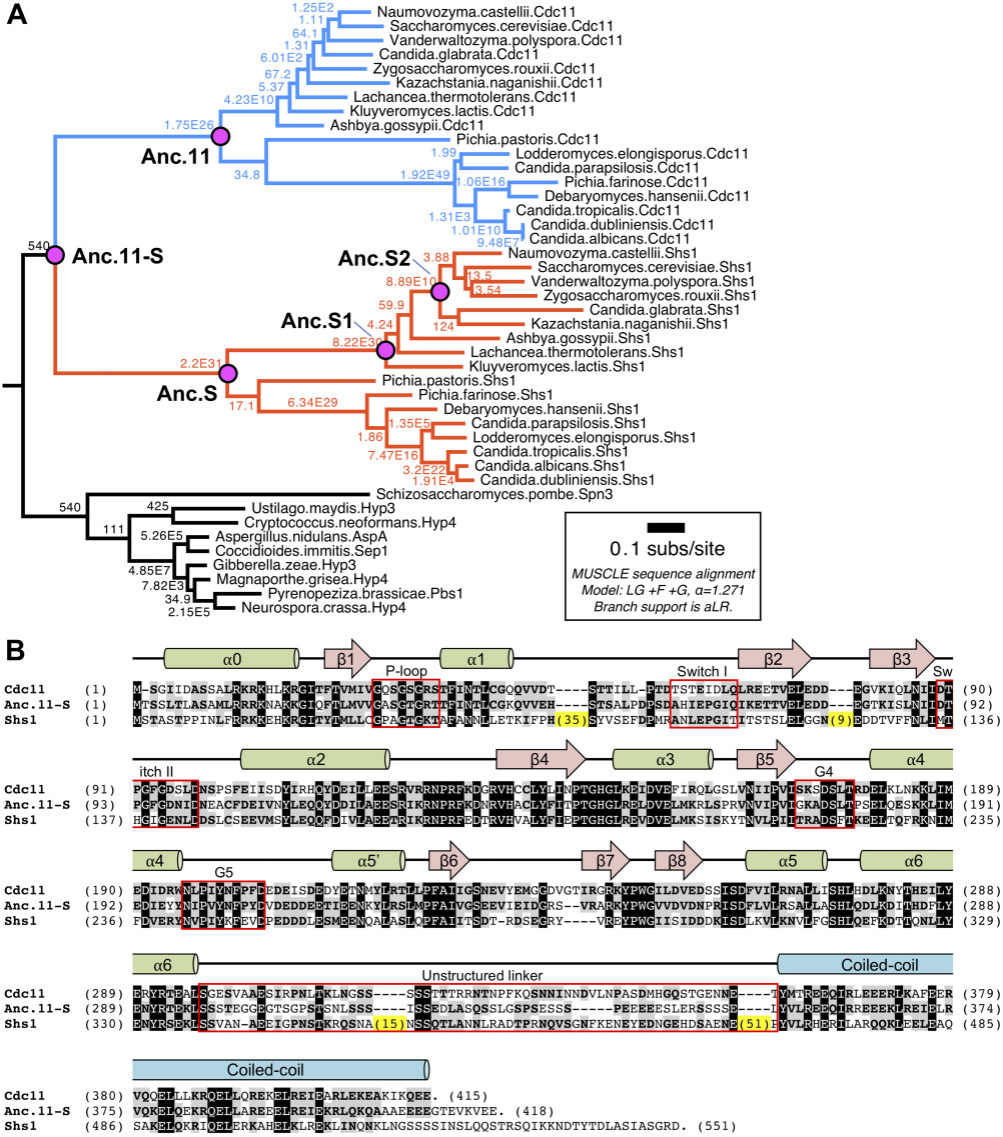
Phylogeny of septins Cdc11 and Shs1 within fungal lineage. (A) Protein sequences were identified using BLAST (NCBI) and either *S. cerevisiae* Cdc11 or *S. cerevisiae* Shs1 as a query sequence. Septin proteins used can be found in Supplementary Table S1. Sequences were aligned using MUSCLE (Edgar 2004). Branch support expresses approximately likelihood ratio test statistics (Anisimova and Gascuel 2006; Anisimova *et al.* 2011), interpreted as the ratio increase in model support for the existence of the branch relative to the next-best model in which the branch does not exist. The Cdc11 lineage is colored in blue, whereas the Shs1 lineage is colored in orange. The position of five reconstructed ancestral proteins is noted. (B) Alignment of budding yeast Cdc11 (top) and Shs1 (bottom), and their predicted common Anc.11-S progenitor (middle), using CLUSTAL W (Thompson *et al.* 1994). Identities (white letter in a black box) among all three, and similarities (bold letter in a gray box) where two of the three are identical or share standard conservative substitutions, as well as inserts (yellow) of the indicated length (number of residues in parentheses) present within Shs1, are indicated. Above the alignment are structural elements, based on (1) the crystal structure of an N- and C-terminally truncated version of *S. cerevisiae* Cdc11 (residues 20-to-298) determined at ∼3 Å resolution (Brausemann *et al.* 2016); (2) mammalian SEPT2 (Sirajuddin *et al.* 2007; Sirajuddin *et al.* 2009) because the Cdc11 structure was solved by molecular replacement and refined using the crystal structure of SEPT2 as the model; and (3) prior sequence alignments, structural predictions, and mutational analysis of both Cdc11 and Shs1 (Versele *et al.* 2004; Versele and Thorner 2004; Finnigan, Booth, *et al.* 2015; Finnigan, Takagi, *et al.* 2015). Septins possess sequence elements required for GTP binding that are conserved among all members of the Ras-related super-family (highlighted within red boxes), dubbed the P-loop (G1), Switch I (G2), Switch II (G3), G4 and G5 motifs (Sprang 1997; Wittinghofer and Vetter 2011).

From available fungal genome sequences, orthologs of Cdc11 or Shs1 were collected (Supplementary Table S1) and a phylogeny was constructed using the parameters and algorithms in MUSCLE (Edgar 2004) (Figure 1A). Fungal septins closely related to, but distinct from, Cdc11 and Shs1 served as an outgroup. Three prediction programs: MUSCLE (Edgar 2004), MSAprobs (Liu *et al.* 2010), and PRANK (Loytynoja and Goldman 2005, 2008) then were used to deduce a pre-duplication ancestor, dubbed Anc.11-S, and the resulting inferred sequences were compared (Supplementary Figure S1). All three programs provided an overall consensus sequence for Anc.11-S with the major differences within the CTE and lacking, in particular, the inserts in the G domain that are present in modern Shs1 (Figure 1B). Indeed, most of the apparent Shs1 counterparts in other fungi have no (or only much smaller) insertions at these positions. Hence, parsimony suggests that these inserts were absent initially and acquired during the evolutionary trajectory toward modern *S. cerevisiae* Shs1, as will be discussed later.

We chose the Anc.11-S deduced by MUSCLE as representative of the most likely common ancestor for several reasons: (1) the total length of the predicted protein (418 residues) was the longest of the three (Supplementary Figure S1) and (2) it lacked gaps within its predicted CTE (Supplementary Figure S1). In the same way, we also predicted, constructed, and studied a likely, most recent common ancestor to all Cdc11-like subunits (Anc.11) and a likely, most recent common ancestor to all Shs1-like subunits (Anc.S), as well as two likely intermediates (Anc.S1 and Anc.S2) within the lineage leading to modern budding yeast Shs1 (Table 1, Supplementary Tables S2 and S3, and Supplementary Figure S3).

Alignment of Anc.11-S with *S. cerevisiae* Cdc11 and Shs1 revealed that 29% of the predicted ancestral residues are retained in both modern *S. cerevisiae* subunits and that 50% of the residues in the predicted ancestor are identical or similar to at least one of those modern subunits (Figure 1B). As noted above, compared to either the predicted Anc.11-S or modern *S. cerevisiae* Cdc11, modern *S. cerevisiae* Shs1 has some extended loops, two within its G domain and two within its CTE (Figure 1B). With regard to the former, our predictive analysis suggests that the origins of the 35-residue insertion after position 41 first appeared early in the trajectory (Anc.S) to modern *S. cerevisiae* Shs1 (Supplementary Figure S3). With regard to the latter, our prior mutational analysis (Finnigan, Takagi, *et al.* 2015) has already demonstrated that the inserts found in the CTE of Shs1 (prior to the predicted CC region) are not required for its unique functions *in vivo*.

The approaches used here for AGR provide a confidence metric (PP) for each predicted residue. For the residues in Anc.11-S, 67% were predicted with a confidence level of 0.60 or higher; and similar levels were found for each of the other deduced ancestral sequences (Supplementary Table S2). Visualization of these confidence levels across all residues for each ancestral protein (Supplementary Figure S4) revealed a number of common patterns: (1) poorly supported residues at the extreme N-terminus of each ancestor; (2) poorly supported residues within the segment of CTEs that are most proximal (and presumably just serving as a linker) to the G domain; and (3) in the lineage toward Shs1, poorly supported residues at two sites within the G domain corresponding to the position of inserts, such as the 35-residue loop in modern *S. cerevisiae* Shs1. Importantly, however, these patterns also include the appearance through evolutionary time of strongly supported residue clusters. For example, residues poorly supported at the extreme C-terminal ends of Anc.11-S and Anc.11 acquire substantial and strongly conserved appendages diagnostic of the Shs1 lineage (Supplementary Figure S4).

### Anc.11-S is able to partially replace modern yeast Cdc11

**Figure 2:**
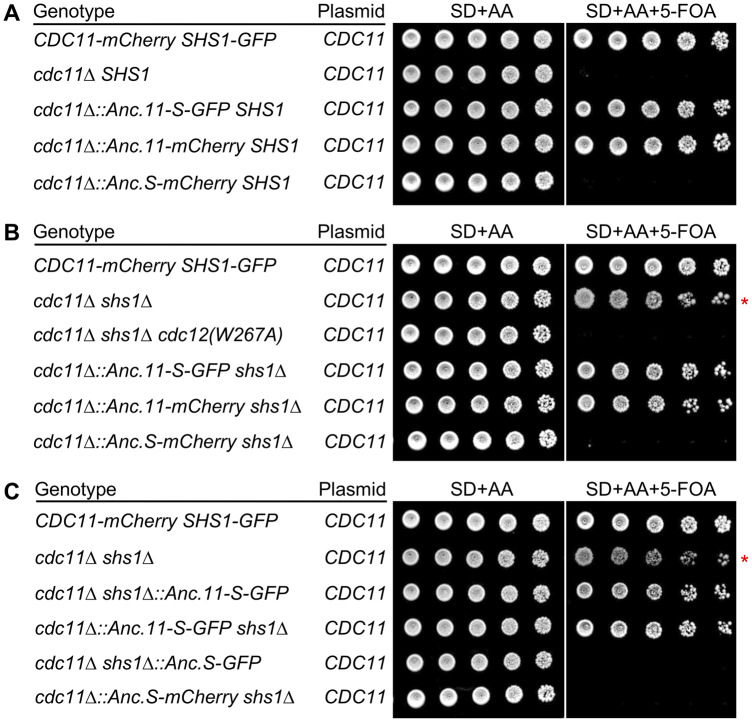
Complementation tests of the ability of three predicted ancestral septins to functionally substitute for modern *S. cerevisiae* Cdc11 and Shs1. (A) Ability of Anc.11-S, Anc.11, or Anc.S to rescue the inviability of *cdc11Δ SHS1* cells. Strains GFY-160, GFY-153, GFY-479, GFY-481, and GFY-975 (all initially harboring a *URA3*-marked covering plasmid expressing WT *CDC11*) were cultured in SD-URA medium overnight at 30°C and serial diluted (fivefold) onto SD+AA or SD+AA+5-FOA agar plates, as indicated, and incubated for 3 days before imaging. (B) Ability of Anc.11-S, Anc.11, or Anc.S to maintain the viability of *cdc11Δ shs1Δ* cells. Strains GFY-160, GFY-163, GFY-437, GFY-480, GFY-482, and GFY-974 were tested as in (A). Red asterisk, abnormal growth of *cdc11Δ shs1Δ* cells (GFY-163) is reflected in altered spot morphology (see also Supplementary Figure S6). (C) Promoter and locus for Anc.11-S, or Anc.S expression does not alter phenotype. Strains GFY-160, GFY-163, GFY-483, GFY-480, GFY-476, and GFY-974 were assayed as in (A). SD, synthetic drop-out medium with dextrose.

DNA encoding an optimized version (*i.e.* using modern *S. cerevisiae* codon usage bias) for each predicted ancient septin was synthesized *de novo*, C-terminally tagged in-frame with the coding sequence for either mCherry or GFP, cloned under control of the natural *CDC11* or *SHS1* promoter, and inserted and expressed from the corresponding native chromosomal locus in place of the endogenous gene in *S. cerevisiae*. We first examined whether or not integrated copies of Anc.11-S, Anc.11, or Anc.S could substitute for the function of modern *S. cerevisiae* Cdc11 (Figure 2). Budding yeast lacking Cdc11, but expressing Shs1, is inviable (McMurray *et al.* 2011; Finnigan, Takagi, *et al.* 2015); hence, in all cases, for these strain constructions and growth assays, the Cdc11 deficiency was covered by a *URA3*-marked plasmid expressing wild-type (WT) *S. cerevisiae CDC11* to maintain viability. The capacity of each construct to support growth could then be tested by selecting for loss of the *URA3*-marked plasmid on medium containing 5-FOA (Boeke *et al.* 1984). Serial dilutions of the strains to be tested were spotted onto either a permissive medium or a medium containing 5-FOA. The cells expressing Cdc11-mCherry and Shs1-GFP (positive control) remained viable in the absence of the *CDC11* plasmid, whereas the *cdc11Δ SHS1* strain (negative control) was inviable when the *CDC11* plasmid was absent, as expected (Figure 2A, lanes 1 and 2). Like cells expressing Cdc11-mCherry, we found that cells expressing either Anc.S-11-GFP or Anc.11-mCherry were viable in the absence of the *CDC11* plasmid (Figure 2A, lanes 3 and 4), whereas cells expressing Anc.S-mCherry were unable to grow (Figure 2A, lane 5). Thus, Anc.11-S and Anc.11 (but not Anc.S) were able to substitute for modern *S. cerevisiae* Cdc11 based on this growth assay. We also examined the cell morphology of strains harboring either Anc.11-S or Anc.11 in place of yeast Cdc11. Compared to a WT strain expressing an integrated copy of Cdc11-mCherry, cells expressing either ancestral subunit appeared similar in shape and size (Supplementary Figure S5). However, there was a subpopulation that appeared to have elongated cell morphologies suggesting that Anc.11-S or Anc.11 cannot provide a full replacement of modern Cdc11 (Supplementary Figure S5). Nonetheless, we find it quite remarkable that this predicted ancient progenitor possesses the capacity to interface with modern septins sufficiently well to maintain viability, support a normal growth rate, and exhibit near-normal morphology in the majority of the cells.

To determine whether the presence of Shs1 contributed to the ability of either Anc.11-S or Anc.11 to function in place of Cdc11, we also tested the same three ancestral subunits in a strain lacking both *CDC11* and *SHS1*. Previous work (McMurray *et al.* 2011) found that cells carrying a *cdc11Δ shs1Δ* double deletion, rather than being inviable, are able to grow, albeit more slowly than normal cells and with an aberrant, markedly elongated and branched morphology, which also manifests at the macroscopic level as an altered colony morphology (Supplementary Figure S6). We were able to readily reproduce those findings (Figure 2B, lane 2). The explanation for the viability of cells lacking both Cdc11 and Shs1 is that the remaining septin hetero-hexamers are still able to form rudimentary filaments via a non-native Cdc12–Cdc12 G interface association (McMurray *et al.* 2011). When only Shs1 is present, it binds to Cdc12, forming hetero-octamers, but Shs1 is unable to self-associate via an NC interface (McMurray *et al.* 2011; Booth *et al.* 2015; Finnigan, Takagi, *et al.* 2015); hence, no filaments can assemble and the cells are inviable. In contrast, when only Cdc11 is present, it binds to Cdc12, restoring hetero-octamer formation *and* mediating filament assembly via a robust Cdc11–Cdc11 NC interface, and thus the cells are viable (Garcia *et al.* 2011; McMurray *et al.* 2011). The non-native homotypic interaction between Cdc12-capped hetero-hexamers can be prevented by a mutation (W267A) that disrupts the G interface (McMurray *et al.* 2011), and we confirmed that *cdc12Δ shs1Δ* cells carrying a *cdc12(W267A)* allele are indeed inviable (Figure 2B, lane 3). Most importantly, we found that, in cells lacking both Cdc11 and Shs1, expression of either Anc.11-S or Anc.11 was able to support normal growth (Figure 2B, lanes 4 and 5), as would be expected for authentic *S. cerevisiae* Cdc11 and with a spot morphology resembling that of the control cells (Figure 2B, lane 1) rather than that of the *cdc11Δ shs1Δ* cells (Figure 2B, lane 2). These findings demonstrate that Anc.11-S and Anc.11 can partially replace yeast Cdc11 in either the presence or absence of modern Shs1. Equally as telling, we found that the expression of Anc.S-mCherry in *cdc11Δ shs1Δ* cells still resulted in no growth (Figure 2B, lane 6), as might be expected for authentic *S. cerevisiae* Shs1. At the very least, this result indicates that the Anc.S subunit is, in fact, produced and must associate with Cdc12, thereby preventing any homotypic Cdc12–Cdc12 interaction. If Anc.S were not actually produced, or not properly folded, or failed to interact with Cdc12, then these cells would have been viable, like the *cdc11Δ shs1Δ* strain itself.

To ensure that the observed results were not influenced by the choice of promoter used for expression (as there is no predicted ancient promoter sequence), we tested the functions of Anc.11-S and Anc.S, each driven by either the *CDC11* promoter or the *SHS1* promoter at their native genomic loci. Regardless of their mode of expression, identical results were obtained for both proteins when each ancestral subunit was expressed in *cdc11Δ shs1Δ* cells (Figure 2C). Therefore, the stark difference in their observed phenotypes cannot be attributed to any difference in expression due to the promoters used or to the genomic location from which they were produced.

### Anc.S does not possess all of the properties of modern *S. cerevisiae* Shs1

*Saccharomyces cerevisiae* cells lacking Shs1 are viable (Iwase *et al.* 2007; Garcia *et al.* 2011) and, conversely, cells expressing Shs1 as the only available terminal subunit for septin hetero-octamers are inviable (McMurray *et al.* 2011; Finnigan, Takagi, *et al.* 2015). Fortunately, we were able to devise previously three, different “sensitized” genetic backgrounds in which the presence of Shs1 is required for cell survival (Finnigan, Takagi, *et al.* 2015). Thus, despite being “non-essential” in normal *S. cerevisiae* cells, these three reporter strains permitted analysis of functional elements in Shs1 (Finnigan, Takagi, *et al.* 2015), identification of some of its interaction partners (Finnigan, Booth, *et al.* 2015), and inferences about its unique contributions to optimal cell function (Egelhofer *et al.* 2008; Buttery *et al.* 2012; Meseroll *et al.* 2012).

**Figure 3:**
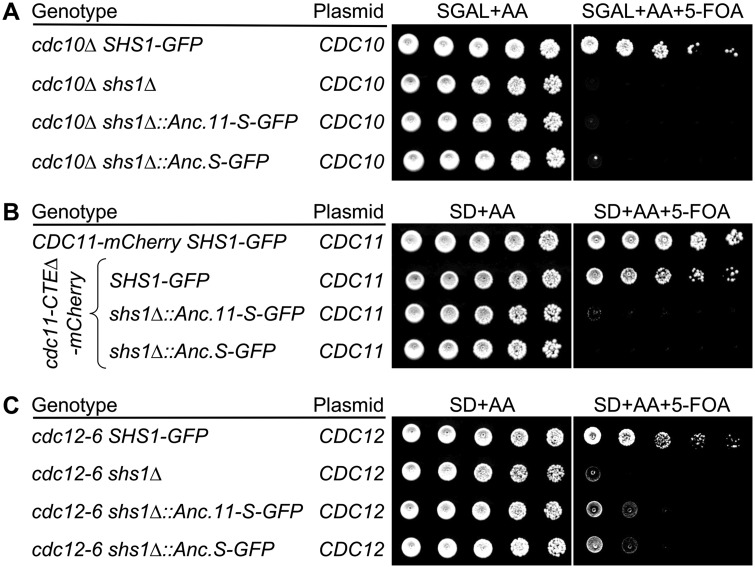
Use of three sensitized backgrounds that require *S. cerevisiae* Shs1 for viability to assess Anc.S function. (A) Ability of Anc.S to support the viability of *cdc10Δ* cells on galactose medium at 22°C. Strains GFY-87, GFY-137, GFY-815, and GFY-760 (all initially harboring a *URA3*-marked covering plasmid expressing WT *CDC10*) were cultured overnight in YPGAL at 22°C, spotted onto plates of SGAL medium in the absence and presence of 5-FOA, as indicated, and incubated at 22°C for 5 days prior to imaging. (B) Ability of Anc.S to support the viability of cells expressing Cdc11(ΔCTE)-mCherry as sole source of Cdc11. Strains GFY-160, GFY-293, GFY-485, and GFY-486 (all initially harboring a *URA3*-marked covering plasmid expressing WT *CDC11*) were grown overnight in SD-URA at 30°C, serial diluted onto plates in the absence and presence of 5-FOA, as indicated, and incubated at 30°C for 3 days. (C) Ability of Anc.S to support the viability of *cdc12-6* mutant cells. Strains GFY-302, GFY-139, GFY-478, and GFY-477 (all initially harboring a *URA3*-marked covering plasmid expressing WT *CDC12*) were grown overnight in SD-URA at 30°C, spotted in the absence and presence of 5-FOA, as indicated, and incubated for 3 days at 22°C.

In the first of these special strains, Cdc10 (the central subunit of septin hetero-octamers) is absent. Under standard laboratory conditions (glucose as the carbon source and 30°C), this strain is inviable. However, prior work showed that on galactose medium at 22°C, *cdc10Δ* cells are able to grow (McMurray *et al.* 2011). The mechanistic explanation, at least in part, for this behavior was determined to be that, under those specific growth conditions, Cdc11–Cdc12–Cdc3 hetero-trimers assemble, associate via a non-native homotypic Cdc3–Cdc3 interaction, and the resulting hetero-hexamers are able to form rudimentary filaments and thereby support growth. However, we found that, in this context, survival of the cells requires the presence of Shs1 (Finnigan, Takagi, *et al.* 2015) (Figure 3A, lanes 1 and 2). In this case, viability during strain construction was maintained by a *URA3*-marked plasmid expressing WT *S. cerevisiae CDC10* and the capacity of any construct to support growth could then be tested by selecting for loss of the *URA3*-marked plasmid on 5-FOA medium. Unlike modern *S. cerevisiae* Shs1, expression of Anc.11-S or Anc.S did not rescue the inviability of *cdc10Δ* cells lacking endogenous Shs1 (Figure 3A, lanes 3 and 4).

The second sensitized genetic background in which we found that presence of Shs1 was essential for viability was in cells expressing a C-terminally truncated *cdc11* allele, Cdc11(Δ357–415), tagged at its C terminus with mCherry as the sole source of Cdc11 (Finnigan, Takagi, *et al.* 2015). In this case, viability during strain construction was maintained by a *URA3*-marked plasmid expressing WT *S. cerevisiae CDC11.* In this context too, unlike modern Shs1 (Figure 3B, lane 2), expression of Anc.11-S or Anc.S was unable to rescue the inviability of the cells expressing Cdc11(Δ357–415)-mCherry (Figure 3B, lanes 3 and 4).

The third background in which the presence of Shs1 is required for normal growth is in cells carrying a temperature-sensitive *cdc12* allele, *cdc12-6*, incubated at what would otherwise be a permissive temperature (22°C) (Figure 3C, lanes 1 and 2). It has been shown elsewhere that although *cdc12-6* cells are able to survive at the lower temperature, they become inviable at a higher temperature (37°C) because their septin filaments disassemble (Johnson *et al.* 2015). In this case, viability during strain construction was maintained by a *URA3*-marked plasmid expressing WT *S. cerevisiae CDC12.* As in the other two sensitized backgrounds, Anc.11-S or Anc.S could not behave like modern *S. cerevisiae* Shs1 (Figure 3C, lanes 3 and 4). Thus, these data indicate that neither of these predicted progenitors (the original pre-duplicated ancestor and the most recent common ancestor to all Shs1-like septins) has yet acquired the full panoply of unique characteristics that define modern Shs1.

### Ancestral septins assemble into the septin collar at the bud neck

**Figure 4:**
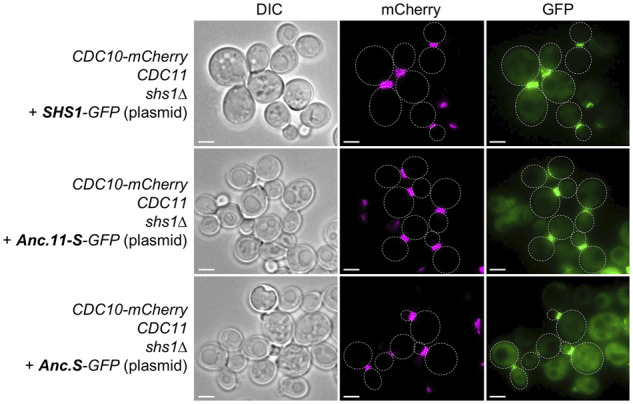
Reconstructed ancestral septins localize to the yeast bud neck congruent with endogenous septins. A *CDC11 shs1Δ* strain (GFY-6) expressing Cdc10-mCherry from the chromosomal *CDC10* locus to mark the location of the septin collar at the bud neck was transformed with plasmids expressing either *S. cerevisiae* Shs1-GFP (pGF-preIVL-59) (top panels), Anc.11-S-GFP (pGF-IVL-159) (middle panels), or Anc.S-GFP (pGF-IVL-168) (bottom panels). The cultures were incubated overnight in SD-LEU at 30°C, back-diluted into YPD, grown for an additional 4.5 h at 30°C, washed with water, and visualized under white light by Nomarski optics (Differential Interference Contrast (DIC), leftmost images) and by fluorescence microscopy with appropriate cutoff filters to detect mCherry (middle images) and GFP (rightmost images), respectively. Representative images, adjusted using ImageJ, are shown. Faint dotted white lines demarcate the cell periphery. Scale bar, 3 μm.

To rule out in an independent way that any lack of functional complementation for any trait examined was due to lack of incorporation of the reconstructed ancestral protein of interest into septin-based structures, we examined localization of Anc.11-S and Anc.S tagged at their C terminus with GFP by live cell imaging using fluorescence microscopy (Figure 4). To mark the location of septin-based structures unequivocally, these cells also expressed an integrated copy of Cdc10-mCherry. To maintain uniform conditions, because expression of Anc.S in cells lacking both Cdc11 and Shs1 does not support growth (Figure 2), we chose to examine expression and localization of these proteins in a *CDC11 shs1Δ* strain. We found that, just like authentic Shs1-GFP (expressed under the *SHS1* promoter on a low-copy plasmid) (Figure 4, top panels), both Anc.11-S-GFP (Figure 4, middle panels) and Anc.S-GFP (Figure 4, bottom panels) localized prominently to the bud neck in dividing cells and completely congruently with the Cdc10-mCherry marker (despite the presence of endogenous Cdc11, which might have been expected to compete with the ancestral proteins for binding to Cdc12). The same pattern was observed for Anc.11-S-GFP and Anc.S-GFP in cells where the septin collar was marked by expression of an integrated copy of Cdc11-mCherry (Supplementary Figure S7). Similarly, in cells where the septin collar at the bud neck was marked with Shs1-GFP, Anc.11-mCherry also localized prominently to the bud neck, even though Cdc11 was also present (Supplementary Figure S7). In the case of Anc.S-GFP, there was a somewhat higher level of diffuse fluorescence in the cytosol than for the other two ancestral proteins (Figure 4 and Supplementary Figure S7). Overall, these observations indicate that all three ancestral proteins are incorporated well into the septin super-structure at the bud neck, and thus are able to compete for occupancy with their modern septin counterparts, presumably because each is able to associate with Cdc12 via their G interface. Moreover, for Anc.S, the collective data up to this point demonstrate that there must be *in vivo* function(s) of Shs1-like septins that are separable from assembly into and localization within the septin collar at the bud neck, as we have documented for modern Shs1 itself (Finnigan, Booth, *et al.* 2015; Finnigan, Takagi, *et al.* 2015).

### Interaction of ancestral septins with extant subunits within and between hetero-octamers

To investigate how Anc.11-S and Anc.11 were participating in contacts within and between septin hetero-octamers, we utilized a previously studied septin allele (Bertin *et al.* 2008) that deletes an alpha helix (α0), corresponding to residues 2–18 in both modern Cdc11 and Shs1, situated just upstream of their G domain. This segment contains residues that participate in contacts important for formation of a fully functional NC interface (Sirajuddin *et al.* 2007; McMurray *et al.* 2011). Shs1 is not essential for growth or filament formation under most conditions, and it has been demonstrated that end-to-end contacts between Cdc11-capped hetero-octamers mediated by formation of homotypic Cdc11–Cdc11 NC interfaces are necessary and sufficient for filament formation both *in vivo* and *in vitro* (Garcia *et al.* 2011; McMurray *et al.* 2011). Thus, when present, how is Shs1 incorporated into the septin super-structure at the bud neck? Because both *in vivo* and *in vitro* studies suggest that homotypic Shs1–Shs1 NC interaction does not occur (McMurray *et al.* 2011; Booth *et al.* 2015; Finnigan, Takagi, *et al.* 2015), one possibility to explain how an Shs1-capped hetero-octamer is assembled into filaments is that heterotypic Shs1–Cdc11 NC junctions can form between the Shs1-capped end of a hetero-octamer and the Cdc11-capped end of another hetero-octamer. In support of this possibility, we have found that when the sole source of Cdc11 is a Cdc11(Δα0) mutant, which perturbs its NC interface, the cells are viable when they also express Shs1, but not when Shs1 is absent or when an Shs1(Δα0) mutant is co-expressed (Supplementary Figure S8A). Thus, homotypic Cdc11(Δα0)–Cdc11(Δα0) interactions alone are too weak to promote sufficient filament formation to maintain viability, whereas Cdc11(Δα0)–Shs1 NC interaction must retain the capacity to do so. Additional support for the role of heterotypic Cdc11–Shs1 interactions in bolstering filament formation is provided by our finding that under the conditions where *cdc10Δ* cells are unable to grow in the absence of Shs1, they are able to grow when Shs1(Δα0) is present (Supplementary Figure S8B), presumably because, like Cdc11(Δα0)–Shs1 interaction, the reciprocal Cdc11–Shs1(Δα0) interaction retains some ability to promote filament formation and/or stability.

**Figure 5:**
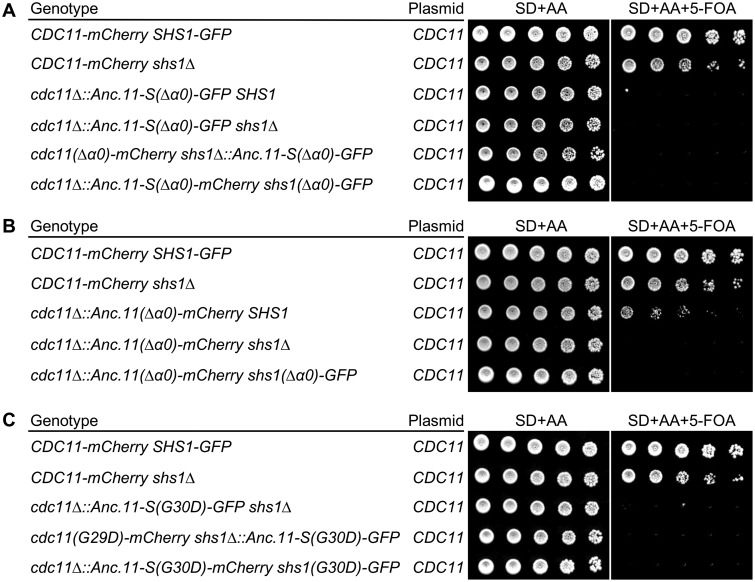
Complementation tests of the functionality of the NC and G interfaces of Anc.11-S and Anc.11. (A) Evidence that Anc.11-S can form a G interface with Cdc12 but cannot form a heterotypic NC interface with Shs1. Strains GFY-160, GFY-164, GFY-566, GFY-660, GFY-564, and GFY-1025 (all initially harboring a *URA3*-marked covering plasmid expressing WT *CDC11*) were cultured in SD-URA at 30°C overnight, serial diluted onto plates in the absence and presence of 5-FOA, as indicated, and incubated at 30°C for 3 days prior to imaging. (B) Evidence that Anc.11 can form both a G interface with Cdc12 and a heterotypic NC interface (albeit weak) with Shs1. Strains GFY-160, GFY-164, GFY-586, GFY-582, and GFY-1023 (all initially harboring a *URA3*-marked covering plasmid expressing WT *CDC11*) were assayed as in (A). (C) Independent confirmation that Anc11-S forms a G interface with Cdc12. Strains GFY-160, GFY-164, GFY-695, GFY-718, and GFY-1026 (all initially harboring a *URA3*-marked covering plasmid expressing WT *CDC11*) were treated as in (A).

These observations provided a means to examine whether Anc.11-S or Anc.11 was able to form a heterotypic junction with Shs1 when present as the sole source of a Cdc11-like septin. Again, as before, viability during strain construction was maintained by a *URA3*-marked plasmid expressing WT *S. cerevisiae CDC11.* We found that, unlike cells expressing modern Cdc11 (Figure 5A, lanes 1 and 2), cells expressing Anc.11-S(Δα0) were inviable in both the presence and the absence of *SHS1* (Figure 5A, lanes 3 and 4), as well as when co-expressed with either Cdc11(Δα0) or Shs1(Δα0) (Figure 5A, lanes 5 and 6). These findings indicated that, unlike modern yeast Cdc11, the pre-duplicated ancestor does not form (productive) heterotypic Anc.11-S–Shs1 interactions *in vivo*, despite its capacity to form homotypic Anc.11-S–Anc.11-S junctions, as reflected in its ability to substitute for modern Cdc11 in the absence of Shs1 (Figure 2). Telling, however, Anc.11(Δα0) tested in the same way was able to support weak, but readily detectable, growth when Shs1 was present (Figure 5B, lane 3), but not when it was absent (Figure 5B, lane 4) or when paired with Shs1(Δα0) (Figure 5B, lane 5). This observation suggests that early on the trajectory from the pre-duplication ancestor to modern Cdc11, the capacity to form a heterotypic junction with a Shs1-like counterpart emerged.

Previous studies (Nagaraj *et al.* 2008; Weems *et al.* 2014) have demonstrated that a G29D mutation in the P-loop of the G domain of Cdc11 (Supplementary Figure S9) weakens a contact important for formation of a fully functional G interface between Cdc11 and Cdc12. This perturbation does not prevent Cdc11 recruitment to the end of a hetero-octamer under normal growth conditions (≤30°C), but does compromise this contact and likely the overall structure of Cdc11 sufficiently to cause cells containing this allele to be inviable at high temperature (37°C). Indeed, we have demonstrated previously that even at permissive temperature, and unlike a *cdc11Δ shs1Δ* strain, *cdc11Δ shs1Δ* cells expressing Cdc11(G29D) are inviable (Finnigan, Takagi, *et al.* 2015), suggesting that Cdc11(G29D) is still able to cap the end of a hetero-octamer, but has such an altered conformation that it is unable to form normal homotypic Cdc11–Cdc11 NC interfaces. In marked contrast, *cdc11Δ shs1Δ* cells expressing the equivalent variant of Shs1, Shs1(G30D), are viable (Finnigan, Takagi, *et al.* 2015), indicating that this septin is unable to associate with Cdc12.

These observations provided a basis to test whether a derivative of Anc.11-S carrying the equivalent mutation, Anc.11-S(G30D), would likewise still retain the capacity to associate with Cdc12 in the hetero-hexamers present in *cdc11Δ shs1Δ* cells and thus behave more like modern Cdc11, or be unable to associate with Cdc12 and thus behave more like modern Shs1. We found that *cdc11Δ shs1Δ* cells expressing Anc.11-S(G30D) were indeed inviable (Figure 5C, lane 3). When Anc.11-S(G30D) was paired with either Cdc11(G29D) or Shs1(G30D), the strains remained inviable (Figure 5C, lanes 4 and 5), as expected.

### Evolution of the G domain and CTE of Shs1

Four of the five mitotically expressed yeast septins (excluding Cdc10) contain prominent CTEs whose sequences each contain a presumptive alpha-helical segment with a strongly predicted propensity to form a CC (Barth *et al.* 2008; Meseroll *et al.* 2012; Sala *et al.* 2016). Previous work (Versele *et al.* 2004; Bertin *et al.* 2008, 2010) has shown that the CTEs of Cdc3 and Cdc12 form a parallel CC that helps stabilize hetero-octamers and also forms an anti-parallel four-helix bundle with its counterpart in a neighboring filament to form the cross-bridges responsible for filament pairing. Along these lines, we showed previously (Finnigan, Takagi, *et al.* 2015) that neither Cdc3 nor Cdc12 could tolerate deletions of the linker region in their CTE that separates their CC from their G domain, whereas both Shs1 and Cdc11 were able to endure large deletions of the corresponding regions [*e.g.* Shs1(Δ342-436) and Cdc11(Δ301-357)] and still retain full function *in vivo*. Even more strikingly, we previously demonstrated (Finnigan, Takagi, *et al.* 2015) that the CTEs of Shs1 and Cdc11 could be swapped; expression of a chimera between the G domain of one terminal subunit fused to the CTE of its paralog allowed for retention of the function of that CTE and subsequent growth, but not if the same CTE was appended to the central subunit Cdc10. Thus, the function(s) of the CTEs of Cdc11 and Shs1 are separable and able to function “*in trans*,” as long as they are located at the terminal end of a hetero-octamer.

The CTE of modern Shs1 is the longest of any of the four extant *S. cerevisiae* septins that have a CTE. To study the evolution of modern yeast Shs1, we generated strains expressing (1) a full-length septin of interest, (2) a chimera between the G domain of *S. cerevisiae* Shs1 and the CTE of our predicted ancestral septins (or from a different extant fungal species), and (3) the reciprocal fusion between the CTE of *S. cerevisiae* Shs1 and the G domain of a different subunit. For existing modern Shs1-like septins from other fungal species, we chose *Candida glabrata, Ashbya* (now *Eremothecium*) *gossypii* and *Candida albicans* (Supplementary Table S3). Each construct was integrated into either a *cdc10Δ shs1Δ* strain (covered initially by a *URA3*-marked *CDC10* plasmid) or a *cdc11Δ shs1Δ* strain (covered initially by a *URA3*-marked *CDC11* plasmid) and expressed from the native *SHS1* promoter at its normal chromosomal locus. Together, these 46 strains provided information and insight on the ability of each construct to associate with Cdc12, form homotypic interactions between hetero-octamers, and exhibit the unique properties that are attributable to modern Shs1 in budding yeast and the degree of functional divergence of the apparent Shs1 orthologs in other distant yeast species.

**Figure 6:**
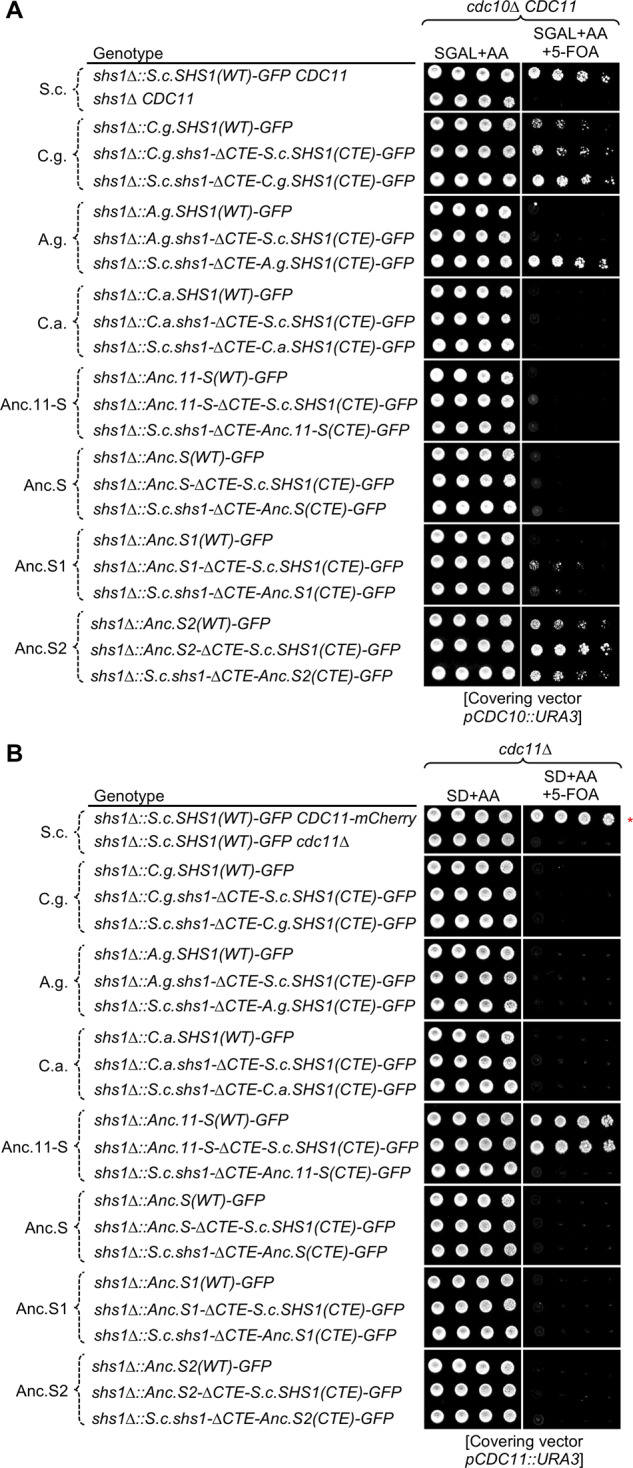
Analysis of the Shs1-like functions of apparent Shs1 paralogs from three distantly related yeast species and four predicted ancestral intermediates in the lineage to modern *S. cerevisiae* Shs1. (A) The sensitized Shs1-dependent *cdc10Δ* background was used to assess the properties of Shs1-like gene products from *Candida glabrata* (*C.g.*), *Ashbya gossypii* (*A.g.*) and *Candida albicans* (*C.a.*) and Anc.11-S, Anc.S, Ans.S1, and Anc.S2 (see Figure 1A). Strains GFY-87, GFY-137, GFY-931, GFY-948, GFY-935, GFY-643, GFY-655, GFY-644, GFY-940, GFY-949, GFY-936, GFY-815, GFY-650, GFY-881, GFY-760, GFY-653, GFY-869, GFY-763, GFY-862, GFY-879, GFY-765, GFY-867, and GFY-868 (all initially harboring a *URA3*-marked covering plasmid expressing WT *CDC10*) were cultured overnight in YPGAL medium at 22°C, spotted onto plates in the absence and presence of 5-FOA, as indicated, and incubated for 5 days at 22°C before imaging. (B) Expression in a *cdc11Δ shs1Δ* strain background was used to assess the Shs1-like properties of the same gene products as in (A). Strains GFY-160, GFY-147, GFY-483, GFY-584, GFY-860, GFY-476, GFY-583, GFY-864, GFY-939, GFY-878, GFY-893, GFY-944, GFY-876, GFY-874, GFY-683, GFY-639, GFY-637, GFY-943, GFY-925, GFY-929, GFY-926, GFY-922, and GFY-938 (all initially harboring a *URA3*-marked covering plasmid expressing WT *CDC11*) were grown overnight in SD-URA at 30°C, spotted in the absence and presence of 5-FOA, as indicated, and incubated for 3 days at 30°C. Red asterisk, the WT strain (GFY-160, lane 1) includes an integrated copy of *CDC11-mCherry* as a positive control. Strains harboring *A. gossypii* constructs were included for a complete comparison to other extant and ancestral subunits; these were tested in a previous study (Finnigan, Takagi, *et al.* 2015).

Expression of these constructs in the sensitized *cdc10Δ* background (on galactose medium at 22°C), which is inviable in the absence of Shs1 (Figure 3A and Figure 6A, lanes 1 and 2), was tested to determine whether any of them was capable of fulfilling the functions of current-day *S. cerevisiae* Shs1. Turning first to extant Shs1 orthologs, *C. glabrata* Shs1 was able to support only very weak growth when present in place of *S. cerevisiae* but functioned significantly better when its own CTE was replaced with the CTE of *S. cerevisiae* Shs1 (Figure 6A, lanes 3 and 4). Revealing, the most robust rescue was observed when the CTE of *S. cerevisiae* Shs1 was replaced with the CTE of *C. glabrata* (Figure 6A, lane 5). Taken together these results suggest that the CTE of *C. glabrata* is functionally equivalent to that of the CTE of endogenous Shs1, and the poor ability of *C. glabrata* Shs1 to complement on its own is likely due to its G domain does not form (1) productive contacts with *S. cerevisiae* Cdc12 and/or (2) optimal NC interface contacts with *S. cerevisiae* Cdc11 between neighboring hexamers in *cdc10Δ* yeast. Similarly, as we have previously documented (Finnigan, Takagi, *et al.* 2015), when the CTE of *A. gossypii* Shs1 was substituted for the CTE of Shs1, it supported vigorous growth, whereas neither *A. gossypii* Shs1 alone nor when the CTE of *A. gossypii* Shs1 was replaced with that from *S. cerevisiae* could do so (Figure 6A, lanes 6–8). Thus, when conveyed to *S. cerevisiae* Cdc12 via the G domain of *S. cerevisiae* Shs1, the CTE of *A. gossypii* clearly could supply near-normal Shs1 function, but its own G domain has lost this ability. The most extreme case we examined was the apparent Shs1 ortholog from *C. albicans* (Figure 6A, lanes 9–11); it is clear that the *C. albicans* CTE does not contain the characteristic functions of *S. cerevisiae* Shs1.

Turning to the predicted ancestral proteins, we found that, with regard to behaving like *S. cerevisiae* Shs1, neither Anc-11.S nor Anc.S had the capacity to do so (Figure 6A, lanes 12–17), akin to the *C. albicans* Shs1 ortholog. In contrast, although Anc.S1 itself could not maintain cell viability, when the CTE of Anc.S1 was replaced with the CTE of modern *S. cerevisiae* Shs1, some very poor, but reproducible, growth was observed (Figure 6A, lanes 18 and 19), suggesting a gradual shift away from the Anc.S identity. Moreover, even when brought to *S. cerevisiae* Cdc12 by the G domain of *S. cerevisiae* Shs1, it is clear that the CTE of Anc.S1 has not acquired modern functionality (Figure 6A, lane 20). In distinct contrast, Anc.S2 itself was able to complement the loss of *S. cerevisiae* Shs1 rather well (Figure 6A, lane 21), even slightly better than the *C. glabrata* Shs1 ortholog, and its CTE has acquired, at least partially, the functionality of the CTE of modern *S. cerevisiae* Shs1 (Figure 6A, lanes 22 and 23). Thus, by these criteria, the fully functional roles of budding yeast Shs1 seem to have arisen rather recently in the evolution of modern *S. cerevisiae* Shs1.

Expression of each of the constructs as a source of Shs1 in the *cdc11Δ* background (Figure 6B) assessed whether any subunit was able to associate with extant Cdc12 *and* mediate sufficient filament formation to maintain viability. Of the 21 proteins tested, only full-length Anc.11-S and Anc.11-S in which its CTE was replaced by the CTE of modern *S. cerevisiae* Shs1 supported growth (Figure 6B, lanes 12 and 13). However, there was a subtle difference in the colony morphology between these two strains: yeast expressing the Shs1 CTE replacement on Anc.11-S appeared to have a rougher colony edge, but not as pronounced as *cdc11Δ shs1Δ* yeast (Figure 2). This may result from the inability of the modern Shs1 CTE domain to contribute to assembly and/or function within hetero-octamers capped exclusively by the Anc.11-S subunit. Thus, the G domain (residues 1–301) of the pre-duplication progenitor possesses the capacity to form a G interface with extant Cdc12 and to self-associate via homotypic Anc.11-S–Anc.11-S NC interfaces to promote the assembly of Anc.11-S-capped hetero-octamers into filaments (and its CTE may be dispensable for these functions). As we have previously observed, strains expressing *A. gossypii* constructs (Finnigan, Takagi, *et al.* 2015) or any other extant or ancestral septin subunit (Figure 6B) were unable to maintain cell viability in this genetic background. However, it remains unclear whether there are differences in gene expression, protein stability, or assembly within the octamer for these constructs that may explain the inability to form functional septin filaments. Together, these findings provide another piece of independent evidence indicating that the capacity to mediate homotypic NC association seems to have been lost very early on in the divergence of Shs1 from Cdc11.

### Overexpression reveals differential affinities for septin-Cdc12 G interface formation

Expression of a protein or any of its variants from an endogenous promoter at its normal chromosomal locus is the most stringent and physiologically meaningful way in which to test biological function. However, retention of partial function can often be uncovered by examining whether any of the same set of proteins is able to function when overexpressed because, as expected from the Law of Mass Action, the effects of a weakened interface can be overcome by raising the concentration of one of the components, thereby pushing the equilibrium toward complex formation, especially in multi-protein ensembles (Sopko *et al.* 2006).

**Figure 7:**
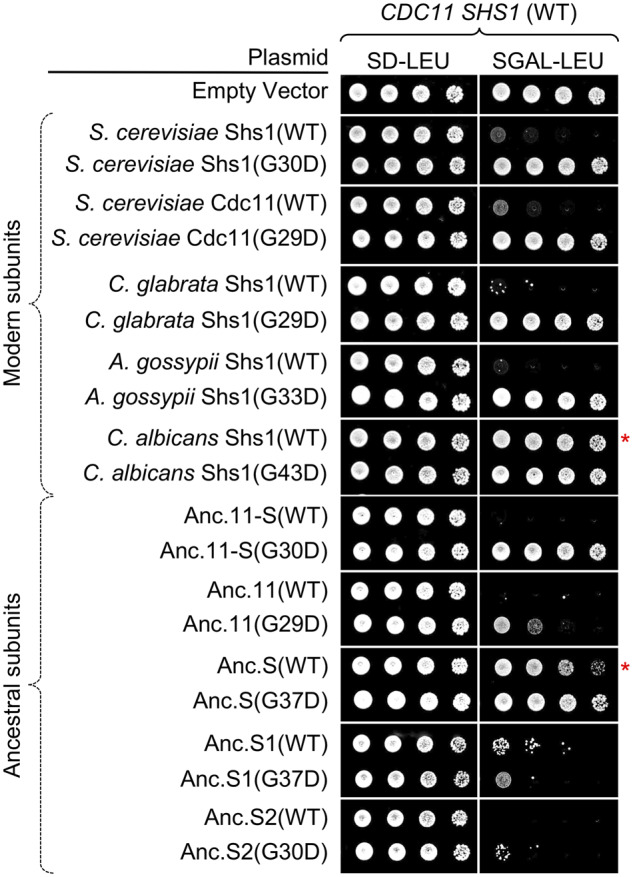
Use of over-expression to assess the capacity for formation of non-native septin interactions. The effects of over-expression driven by the *GAL1/10* promoter of Shs1-like gene products from *Candida glabrata*, *Ashbya gossypii*, and *Candida albicans* and of all five predicted ancestral species constructed in this work (Anc.11-S, Anc.11, Anc.S, Ans.S1, and Anc.S2). An otherwise WT strain (BY4741) was transformed with plasmids pRS315, pGF-IVL-286, pGF-IVL-287, pGF-IVL-1278 through pGF-IVL-1293, pGF-IVL-1343, and pGF-IVL-1344, and cultures of the resulting transformants were grown overnight under non-inducing conditions (S+RAF/SUC-LEU medium) at 30°C, serial diluted onto plates containing either a repressing (D; dextrose/glucose) or an inducing (GAL, galactose) carbon source, as indicated, and incubated for 3 days at 30°C prior to imaging. Growth of constructs marked with a red asterisk was also monitored at 2 and 4 days in several strain backgrounds (Supplementary Figure S10). RAF, raffinose; SUC, sucrose.

A previous dosage screen (Sopko *et al.* 2006) in *S. cerevisiae* had suggested that production of either terminal septin subunit at a very high level was toxic in otherwise normal cells. Indeed, when we overexpressed either Shs1 or Cdc11 in otherwise WT cells using the galactose-inducible *S. cerevisiae GAL1/10* promoter, growth was markedly impeded (Figure 7, lanes 2 and 4). This growth-inhibitory effect requires their ability to form a G interface because it was eliminated by equivalent P-loop mutations in each protein [Shs1(G30D) and Cdc11(G29D)] (Figure 7, lanes 3 and 5). Under these conditions, however, we cannot determine whether the G interface with Cdc12 in question is the cause of the toxicity, or non-native G–G homotypic association of the overproduced septin itself [which is often observed *in vitro*; for review, see McMurray and Thorner (2019)], or one or more unnatural heterotypic G–G associations with a different septin(s) with which it might not normally interact (which has sometimes been observed *in vivo*; Versele *et al.* 2004; McMurray *et al.* 2011). In any event, by this same criterion, the Shs1 orthologs of *C. glabrata* and *A. gossypii* have the capacity to form a G interface, likely with some extant *S. cerevisiae* septin (Figure 7, lanes 6–9), but that the Shs1 ortholog of *C. albicans* does not in the context of otherwise WT yeast expressing both *S. cerevisiae* Cdc11 and Shs1 (Figure 7, lanes 10 and 11 and Supplementary Figure S10). By the same reasoning, among the predicted ancestral subunits, Anc.11-S behaves quite similar to either Cdc11 or Shs1 (Figure 7, lanes 12 and 13), whereas the toxicities of overexpressed Anc.11 (Figure 7, lanes 14 and 15), Anc.S1 (Figure 7, lanes 18 and 19), and Anc.S2 (Figure 7, lanes 20 and 21) likely arise from other causes (*e.g.* aggregation or misfolding, perhaps). By contrast, Anc.S seems to exhibit only a weak capacity for G interface formation (Figure 7, lanes 16 and 17 and Supplementary Figure S10), similar to the *C. albicans* Shs1 ortholog in a strain also expressing WT Shs1 and Cdc11.

## Discussion

Gene duplication events (at multiple scales) are an important source of new material to fuel the evolution of biological systems (Ohno 1970). When examining the evolution of large multimeric protein-based structures in eukaryotes, it is clear that duplication events have increased the number of individual polypeptides that assemble into the fully functional complex or oligomeric enzyme (Magadum *et al.* 2013; Copley 2020). However, this trend might seem counter-productive, in that, in some organisms, a “simpler” version of the same protein complex has an identical function, yet makes do with fewer separate parts (Finnigan *et al.* 2011; Finnigan *et al.* 2012). Therefore, it is critical to understand this common tendency toward increased biological complexity at a detailed mechanistic level.

Septin-based structures in eukaryotes have a deeply rooted evolutionary history and a highly conserved overall organization in the opisthokont lineage from single-celled yeast to humans (Nishihama *et al.* 2011; Auxier *et al.* 2019; McMurray and Thorner 2019). Yet, within any given fungal or mammalian organism (or, in metazoans, cell type), septin hetero-octamers can be assembled from alternative sets of subunits, which, it has been proposed, arose from gene duplication and divergence (Cao *et al.* 2009; Valadares *et al.* 2017). Ostensibly, this diversification has allowed different combinations of subunits associated with a common core structure to generate distinct supramolecular arrangements that fulfill separate physiological functions using the same underlying scaffold (Barral and Kinoshita 2008; Garcia *et al.* 2011, 2016; Vargas-Muñiz *et al.* 2016; Khan *et al.* 2018; Rosa *et al.* 2020).

**Figure 8:**
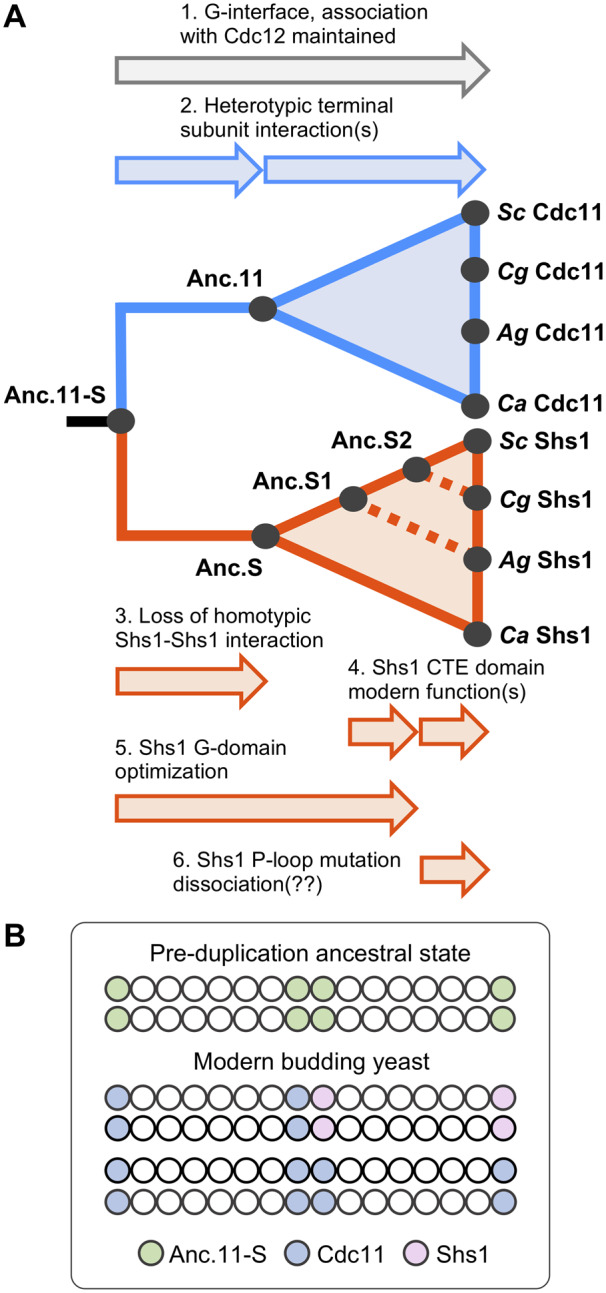
Model for gene duplication and functional divergence in the evolution of the essential septin Cdc11 and its non-essential paralog Shs1 within the fungal clade. (A) A simplified phylogeny of the evolutionary trajectory of the terminal septin subunits, highlighting the findings made in this study. 1. All ancestors and tested modern fungal subunits were able to form a G interface with the penultimate subunit Cdc12 and assemble into the septin hetero-octamer *in vivo*. 2. Anc.11-S was unable to form a heterotypic NC interface with modern Shs1, whereas Anc.11 has acquired to a readily detectable degree the ability to form a heterotypic NC interface with modern Shs1. 3. All Shs1 subunits tested, including Anc.S, were unable to form functional homotypic NC interface interactions. 4. The distinct function(s) of the Shs1 CTE evolved late in the Shs1 lineage, after Anc.S1. 5. An optimal Shs1 G domain appeared in Anc.S2. 6. Modulation of Shs1–Cdc12 association and/or assembly at the G interface appears to occur after Anc.S2. (B) Model of how duplication of Anc.11-S and the ensuing advents of modern Cdc11 and Shs1 allows for an expansion in the repertoire of potential filament-forming complexity.

Septin structures erected during vegetative growth of the budding yeast *S. cerevisiae* (Farkašovský 2020; Marquardt *et al.* 2019) are assembled from two otherwise identical protomers: Cdc11-capped hetero-octamers or Shs1-capped hetero-octamers. Although each is likely symmetric (*i.e.* possessing the same terminal subunit at each of its ends) (Khan *et al.* 2018), the possibility of a mixed hetero-octamer (*i.e.* with Cdc11 at one end and Shs1 at the other) has not been completely ruled out. In this study, we deduced, constructed, and tested the properties of a predicted likely common ancestor (Anc.11-S) of both Cdc11 and Shs1, as well as proposed representatives of likely intermediates on the trajectory to Cdc11 (Anc.11) and to Shs1 (Anc.S, Anc.S1, and Anc.S2). We found that, like modern Cdc11 itself, both Anc.11-S and Anc.11 were able to associate with the penultimate subunit (modern Cdc12) via their G interface and able to maintain cell viability, indicating that they must also self-associate via forming homotypic NC interfaces, thereby mediating polymerization of hetero-octamers into functional filaments. Thus, it appears that the capacity for promoting filament assembly was retained within the Cdc11 lineage (Figure 8A). Preservation of such self–self interactions has been observed in other cases where complexity has increased due to gene duplication and divergence (Pereira-Leal and Teichmann 2005; Pereira-Leal *et al.* 2007).

After duplication of the common ancestor, other potential arrangements (aside from ‘Cdc11’–Cdc12 and ‘Cdc11’–‘Cdc11’) became potential options, namely ‘Shs1’–Cdc12, ‘Shs1’–‘Cdc11’, and ‘Shs1’–‘Shs1’. With regard to the latter possibility, we found that, like modern Shs1 itself, neither Anc.S, Anc.S1, nor Anc.S2 retained the capacity for homotypic association. Hence, it appears that loss of a direct filament-promoting function occurred early in the Shs1 lineage (Figure 8A). However, there was the apparent gain of the capability for subunits in the Shs1 lineage to form a heterotypic ‘Shs1’–‘Cdc11’ NC interface, which obviously would expand the repertoire of higher-order structures achievable, perhaps providing an initial selective advantage for acquisition and fixation of this property.

In contrast to the loss of homotypic NC interface formation, our analysis revealed that, like modern Shs1, Anc.S, Anc.S1, and Anc.S2 (as well as the Shs1 orthologs from three other extant yeast species distant from *S. cerevisiae*) retained the capacity to form a G interface with the penultimate subunit (modern Cdc12), albeit with rather widely different apparent affinities. Of course, it seems reasonable to assume that for all of the predicted ancestral septins tested that, at the same point in the evolutionary trajectory, the Cdc12 equivalent with which they associated likely differed in sequence to varying extents from that of modern *S. cerevisiae* Cdc12. Likewise, we know that the sequences of the Cdc12 partners for the Shs1 orthologs of the extant species tested here also differ in sequence from that of modern *S. cerevisiae* Cdc12 (Pan *et al.* 2007; Nishihama *et al.* 2011). This non-native ancient-to-modern G interface between yeast Cdc12 and the ancient septins may also explain why we observed elongated cellular morphologies in strains expressing Anc.11-S or Anc.11 *in vivo*.

To assess the acquisition of the features that distinguish the unique CTE of modern Shs1 from that of modern Cdc11, we utilized three sensitized genetic backgrounds in which authentic Shs1 must be present for the cells to remain viable. We found that glimmers of the characteristics that distinguish the CTE of modern Shs1 could be observed in Anc.S1 and were much more robustly exhibited by Anc.S2, but only fully displayed by modern Shs1 itself and preserved in orthologs from certain other yeasts (especially *C. glabrata* and *A. gossypii*). Thus, the changes that neo-functionalized Shs1 seem to have occurred in stepwise fashion and emerged rather late in the Shs1 lineage (Figure 8A). Indeed, although modern fungal Shs1 is “non-essential,” it makes readily measurable contributions to optimal cell physiology, such as reinforcing recruitment of certain septin-associated proteins required for cytokinesis (Finnigan, Booth, *et al.* 2015) and phosphorylation-dependent control of the geometries and disassembly dynamics of higher-order septin-based structures (McQuilken *et al.* 2017; Khan *et al.* 2018).

Prior work demonstrated that Cdc12 (and Cdc10) possesses low, but readily detectable, GTPase activity, but Cdc3, Cdc11, and Shs1 do not (Versele and Thorner 2004; Sirajuddin *et al.* 2009); and recent work (Weems and McMurray 2017) indicates that, when GTP-bound, Cdc12 associates preferentially with Cdc11, whereas when GDP-bound, Cdc12 associates preferentially with Shs1, explaining, at least in part, the basis of the differential incorporation of the two different terminal subunits into the corresponding hetero-octamers. Our findings here, while consistent with those conclusions, address how changes during the divergence of the Cdc11 and Shs1 lineages from their common pre-duplication ancestor have contributed to modulating their differential affinities for the formation of a G interface with Cdc12. We found that Anc.11-S and Anc.11, like modern Cdc11, exhibited a robust capacity for binding to Cdc12, whereas during the progression toward modern Shs1, due to cumulative sequence alterations (possibly including numerous insertions in its G domain), the affinity of Shs1 for Cdc12 has been significantly reduced (Figure 8A), in agreement with earlier *in vitro* biochemical results demonstrating that the off-rate for dissociation of Shs1 from purified recombinant Shs1-capped hetero-octamers is substantially higher than for dissociation of Cdc11 from purified Cdc11-capped hetero-octamers (Garcia *et al.* 2011).

In this regard, how high-level overexpression of Cdc11 or Shs1 (but no other septin) is toxic to the growth of otherwise WT yeast cells involves inappropriate capping of hetero-octamer ends, thereby preventing formation of functional filaments (which occurs via polymerization of preformed hetero-octamers; Bridges *et al.* 2014), but the mechanism by which each does so in the cell is distinct. Even though it binds more weakly to Cdc12, excess over-expressed Shs1 outcompetes the level of endogenous Cdc11, resulting in mainly Shs1-capped hetero-octamers, which lack the capacity for homotypic Shs1–Shs1 NC interaction, thereby blocking filament formation, as deduced previously (McMurray *et al.* 2011). By contrast, in the presence of a much greater than stoichiometric level of Cdc11, it is possible that homotypic Cdc11–Cdc11 NC interaction between free Cdc11 monomers and the ends of Cdc11-capped hetero-octamers generates non-natural hetero-decamers that are unable to polymerize via a homotypic Cdc11–Cdc11 G interface. Alternatively, if a homotypic Cdc11–Cdc11 G interface between such non-natural hetero-decamers is able to form, the more extended structure of the resulting filaments must be so aberrant as to preclude viability. We favor somewhat the latter explanation because we observed (Figure 7) that overexpression of the Cdc11(G29D) P-loop mutant, which cripples its G interface (and likely alters conformation sufficiently so as to also weaken its NC interface), completely eliminated its overexpression-based toxicity (Figure 7). Of course, it is also possible that massive overexpression of GTP-bound Cdc11 [but, not “empty” Cdc11(G29D)] titrates out protein chaperones needed for folding of Cdc11 itself, thereby preventing efficient folding of the other septin subunits and other essential cellular proteins (Johnson *et al.* 2015).

The genomes of many species, especially mammals, encode an assortment of alternative septin subunits, which can be differentially expressed in different cell types and further diversified by alternative splicing and other means, allowing for assembly of distinct types of hetero-octamers in specific tissues or during different developmental programs (Hall and Russell 2012; Neubauer and Zieger 2017). It has been unclear; however, the degree to which existing septin assemblies could accommodate predicted ancestral subunit(s) or modern septins that have evolved in extant, but distantly related, species. With regard to the latter point, septins from certain heterologous sources have been tested in *S. cerevisiae*. The apparent Cdc12 ortholog from the filamentous fungus *Aspergillus nidulans* AspC was able to complement the inviability of a *cdc12Δ* mutant only poorly, and when expressed in WT cells, it promoted formation of atypical pseudohyphae rather than normal buds, even though it appeared to localize at the bud neck (Lindsey *et al.* 2010). Recently, the major isoforms of all 13 human septin gene products were tested for their ability to rescue *cdc3Δ*, *cdc10Δ*, *cdc11Δ*, and *cdc12Δ* mutant cells; and only complementation of *cdc10Δ* cells was observed (Garge *et al.* 2020). Of the 13 human septins, only four—two from homology Group 1A (SEPT3 and SEPT9) and two from homology Group 1B (SEPT6 or SEPT10)—were able to exhibit a Cdc10-like function *in vivo* but could not fully replace the yeast subunit (Garge *et al.* 2020). Phylogenetic analysis suggests that human Group 1A and IB septins may share a common ancestor with *S. cerevisiae* Cdc10 (Pan *et al.* 2007). The most recent studies of the human hetero-octamer support an organization (SEPT2–SEPT6–SEPT7–SEPT9–SEPT9–SEPT7–SEPT6–SEPT2) in which a Group1A NC homodimer forms the core of the human septin hetero-octamer (McMurray and Thorner 2019; Mendonca *et al.* 2019; Soroor *et al.* 2020), just as a Cdc10–Cdc10 NC homodimer forms the core of the yeast septin hetero-octamer. So, the partial rescue by SEPT9 (and its paralog SEPT3) of Cdc10 deficiency makes structural sense. By contrast, the rescue of Cdc10-deficient cells by human SEPT6 (and its paralog SEPT10), which occupies the same position in human hetero-octamers as Cdc12 in *S. cerevisiae* hetero-octamers, is harder to explain. Nonetheless, this reported complementation presumably requires that SEPT6 (and SEPT10) be able to form a functional NC homodimer that is able to engage at its flanks yeast Cdc3 via a G interface, highlighting the incredible flexibility inherent in septin–septin interaction. In this same regard, it has been inferred that the (obligate) inclusion of Cdc10 at the central position within the yeast hetero-octamer may have been coupled to the loss of the ability of the Cdc3 subunit to hydrolyze its bound GTP, an event that seems to have occurred prior to the split between the yeast genera *Saccharomyces*, *Ashbya*, and *Kluyveromyce* (Johnson *et al.* 2020). Indeed, biochemical studies of the corresponding human proteins (Zent and Wittinghofer 2014) demonstrate that SEPT9, like yeast Cdc10, is GTPase competent, whereas the flanking septin, SEPT7, like yeast Cdc3, lacks the capacity to hydrolyze its bound GTP.

In conclusion, our study provides the first analysis *in vivo* of predicted intermediates in the evolution of the two paralogs that are able to occupy the terminal position in the septin hetero-octamers of *S. cerevisiae* (Figure 8B). Our findings shed light on why Cdc11 is essential and why Shs1 is not, define the complexities involved in maintaining ancestral protein interactions, and delineate when the various functional features that define and distinguish Cdc11 and Shs1 emerged and diverged. Future work will focus on investigating whether any specific residue change (or small set of residues) is necessary and/or sufficient to recapitulate the steps in the progression from the ancestral state to their modern counterparts.

## Ethical statement

This work did not involve any human or animal subjects of any kind.
